# Photothrombotic Middle Cerebral Artery Occlusion in Mice: A Novel Model of Ischemic Stroke

**DOI:** 10.1523/ENEURO.0244-22.2022

**Published:** 2023-02-07

**Authors:** Emilia Conti, Noemi Carlini, Benedetta Piccardi, Anna Letizia Allegra Mascaro, Francesco Saverio Pavone

**Affiliations:** 1 Neuroscience Institute, National Research Council, 56124 Pisa, Italy; 2 European Laboratory for Non-Linear Spectroscopy, 50019 Sesto Fiorentino, Italy; 3 Neurofarba Department, University of Florence, 50139 Florence, Italy; 4 Department of Physics and Astronomy, University of Florence, 50019 Sesto Fiorentino, Italy; 5 National Institute of Optics, National Research Council, 50019 Sesto Fiorentino, Italy; 6 Translational Research on Stroke (TREES) Working Group, Florence, Italy

**Keywords:** astrocytes, BBB permeability, clasping test, immunofluorescence, MCA photothrombotic occlusion

## Abstract

Stroke is one of the main causes of death and disability worldwide. Over the past decades, several animal models of focal cerebral ischemia have been developed allowing to investigate pathophysiological mechanisms underlying stroke progression. Despite intense preclinical research efforts, the need for noninvasive mouse models of vascular occlusion targeting the middle cerebral artery yet avoiding mechanical intervention is still pressing. Here, by applying the photothrombotic stroke model to the distal branch of the middle cerebral artery, we developed a novel strategy to induce a targeted occlusion of a large blood vessel in mice. This approach induces unilateral damage encompassing most of the dorsal cortex from the motor up to the visual regions 1 week after stroke. Pronounced limb dystonia one day after the damage is partially recovered after one week. Furthermore, we observe the insurgence of blood vessel leakage and edema formation in the peri-infarct area. Finally, this model elicits a notable inflammatory response revealed as a strong increase in astrocyte density and morphologic complexity in the perilesional region of the cortex compared with both other regions of the ipsilesional and contralesional hemispheres, and in sham-operated mice. To conclude, the stroke model we developed induces in mice the light-mediated occlusion of one of the main targets of human ischemic stroke, the middle cerebral artery, free from the limitations of commonly used preclinical models.

## Significance Statement

Cerebral ischemic stroke is one of the leading causes of death and disability worldwide. Animal models represent a fundamental benchmark to investigate the pathophysiological mechanisms underlying the outcomes of stroke patients. Here, we developed and characterized a novel mouse model of stroke using the photothrombotic occlusion of the middle cerebral artery, one of the most common injury sites in stroke patients. The light-mediated occlusion leads in the acute phase to a severe motor deficit accompanied by the insurgence of blood–brain barrier extravasation, and the establishment of an inflammatory regime particularly pronounced in the peri-infarct cortex. This simple and highly reproducible model faithfully recapitulates human ischemic stroke avoiding common drawbacks of other stroke models.

## Introduction

Stroke seriously threatens human health due to its high morbidity, disability, and mortality, thus representing a heavy financial and mental burden affecting families and society (Wafa et al., 2020). Intravenous thrombolysis and endovascular treatment are the standard therapies for patients with acute ischemic stroke. Unfortunately, due to the narrow time window of these treatments, possible treatment inefficacy in terms of recanalization, and the occurrence of reperfusion injury, there is still high variability in determining patient prognoses. These aspects draw the attention of preclinical research aiming to develop animal stroke models to further elucidate the pathophysiological mechanisms of injury and investigate the main processes of neurovascular disruption. In the past few decades, many strategies have been applied to induce ischemic insult in the brain tissue in animal models. Among the several models developed, middle cerebral artery (MCA) occlusion and photothrombosis (PT) are the most diffuse approaches, though they are characterized by some fundamental drawbacks (Macrae, 2011; Conti et al., 2021).

The intraluminal suture of the middle cerebral artery induces damage in the striatum and cortex, generating a sizable volume of penumbra (Carmichael, 2005) with the advantage of avoiding craniotomy and possible brain injury consequent to the surgery (Mies et al., 1991; Menzies et al., 1992). Nevertheless, MCA occlusion procedures are surgically demanding and may induce local traumatic effects (Kanemitsu et al., 2002). Moreover, in this model, the success rate of occlusion and the reproducibility of the infarct size are sometimes unsatisfactory (Yao et al., 2003; Macrae, 2011). On the other hand, the photothrombotic damage shares essential mechanisms occurring with human stroke including the interruption of blood flow caused by platelet aggregation and alterations of the blood–brain barrier (BBB; Dietrich et al., 1987), guaranteeing a high reproducibility between subjects and the capability to easily target the lesioned area (Balbi et al., 2017; Allegra Mascaro et al., 2019). Nevertheless, this method, widely applied to induce small focal lesions, is poorly used in large blood vessels that would better represent severe human infarct. Having said that, a search for an occlusion model that encompasses the broad multiplicity of human ischemic progression is still a challenge for preclinical researchers.

Here, we developed and characterized in elderly mice a novel photothrombotic model of the MCA distal branch. We performed *in vivo* evaluations of mouse behavior and body weight 24 h and 7 d after stroke induction. Then, we quantified the extension of the lesion through *ex vivo* immunostaining. Finally, we characterized BBB permeability 24 h after stroke and alterations of astrocytes morphology through *ex vivo* immunohistochemistry 7 d after PT.

## Materials and Methods

### Mice

All procedures involving mice were performed in accordance with the regulations of the Italian Ministry of Health Authorization number 723/2019. Mice were housed in clear plastic cages under a 12 h light/dark cycle and were given *ad libitum* access to water and food. We used a transgenic mouse line, C57BL/6J-Tg(Thy1-EGFP)MJrs/J, from The Jackson Laboratory. Twenty-nine mice were identified by earmarks and were numbered accordingly. Animals were randomly divided into the following two groups: stroke mice (MCAPT *n* = 15; EB *n* = 6) and sham-operated mice (Sham MCAPT, *n* = 4; Sham EB, *n* = 4). To perform the brain water content evaluation and wire-hanging behavioral test, we used eight mice (Sham, *n* = 4; MCAPT, *n* = 4). Sham-operated mice were subjected to the same surgery and procedure with respect to MCAPT mice except for the Rose Bengal injection, replaced by the injection of the same volume of saline. Each group contained comparable numbers of male and female mice. The age of mice (age range, 16–18 months) was consistent between groups.

### Photothrombotic occlusion of the distal branch of the middle cerebral artery

**Figure 1.**
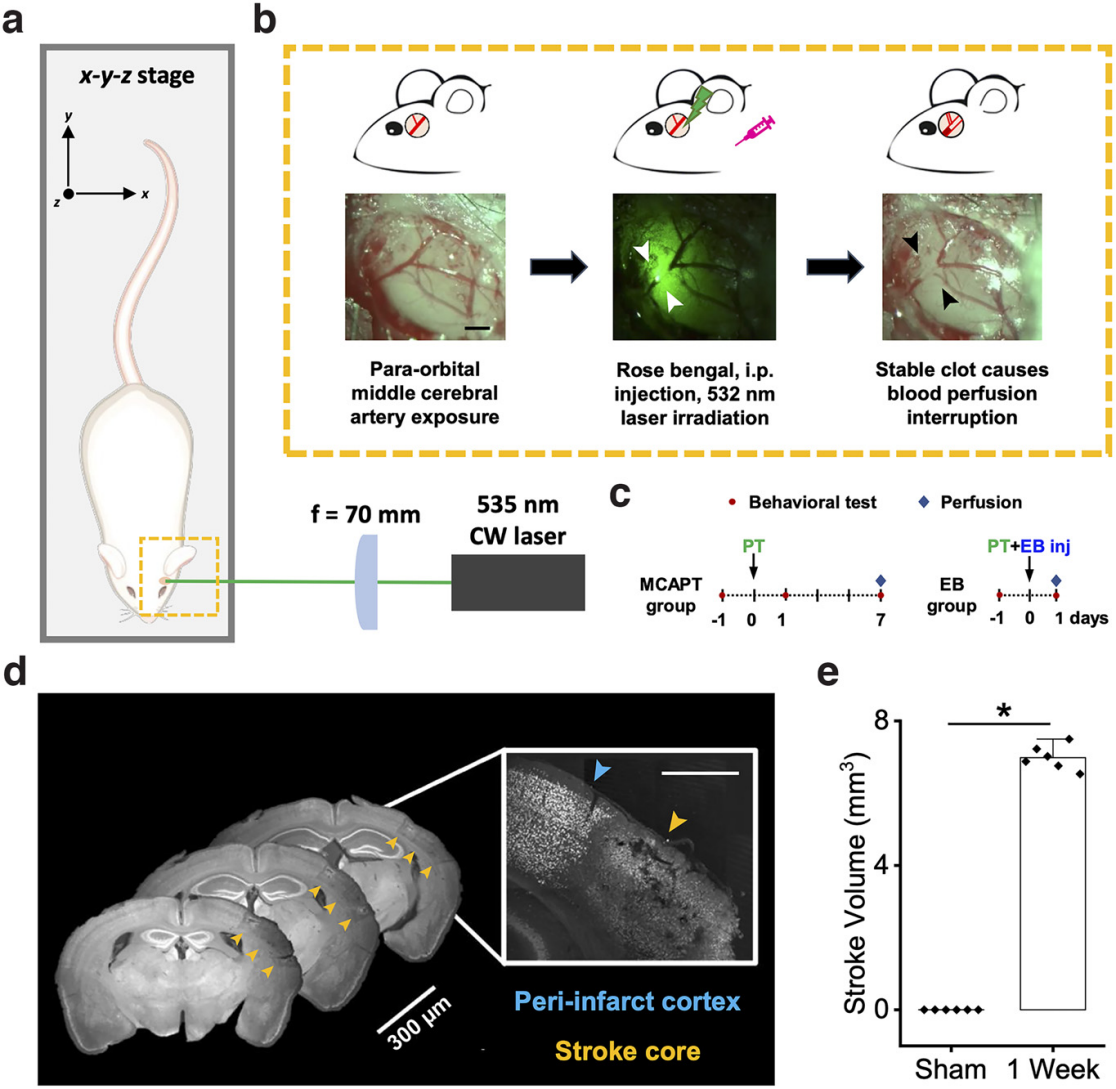
A novel single-vessel mouse model of photothrombotic stroke. ***a***, Representative scheme of the custom-made setup for photothrombosis occlusion of the distal branch of the MCA; for details, see Materials and Methods. ***b***, Representative scheme of the main steps of photothrombotic occlusion of the distal branch of the MCA and corresponding images acquired during surgery. Left, Exposure of the MCA after craniotomy. Middle, Highlighting of the laser irradiation focused on the blood vessel. Right, Formation of the clot. Scale bar, 0.5 mm. ***c***, Experimental timeline for the two groups MCAPT and EB. ***d***, Representative brain slices labeled with NeuN antibody. To quantify the lesion volume, we analyze one slice every 300 μm. The image in the inset, acquired with a confocal microscope, shows a boundary region between the periinfarct cortex and the stroke core. Scale bar, 1.25 mm. ***e***, Right Quantification (mean ± SEM) of stroke volume for the Sham group (0.1 ± 0.0001) and MCAPT group 1 week after photothrombosis (6.9 ± 0.1 mm^3^). *p = 2.29E-08 based on one-way ANOVA followed by a *post hoc* Tukey’s HSD test (*n* = 6). The error bar for the Sham group (*n* = 4) is below the minimum threshold. See also Extended Data Figure 1-1.

Mice were anesthetized with isoflurane (4% induction, 1.5% maintenance, in 1 L/min oxygen). Body temperature was maintained at 37°C with a heating pad (ThermoStar Temperature Controller, RWD). Mice were placed on a surgery pad, lying on one side. To ensure the stability of the animal, the mouse mouth was secured to the incisor bar and then blocked to the surgery pad. The mouse tail was then tightened to the surgery pad. The muscle over the squamosal bone was stretched with surgical tape to ensure more stability during the surgery. The mouse hairs between the eye and the ear were removed and then the skin was cleaned with betadine and ethanol. Then, local anesthetic lidocaine 2% (20 mg/ml) will be applied. The skin over the squamosal bone was cut, and the muscle was detached from the skull and gently pushed down to expose the bone. We used a dental drill (Silfradent) to create a small craniotomy over the squamosal bone to expose the distal branch of the middle cerebral artery. Once removed the flap bone, a photosensitive dye, Rosebengal (0.2 ml, 10 mg/ml solution in Phosphate Buffer Saline (PBS), was intraperitoneally injected (Sigma Aldrich, USA). To induce PT, we developed a custom-made setup to finely controlled the laser irradiation on the distal branch of the middle cerebral artery (Fig. 1*a*). To this aim, we used a 532 nm laser (Laser Diode CPS532, ThorLabs) focused with a 70 mm lens onto the targeted blood vessel. The laser intensity at the focus was 128 mW/mm^2^ (Watson et al., 2002). The mouse was held by the side on a stage, allowing displacements in the *x*-*y*-*z* directions (Translation Stage DTS25/M, Thorlabs).

10.1523/ENEURO.0244-22.2022.f1-1 Figure 1-1 Sham mice *ex vivo* does not show sign of tissue suffering. On the right, panels ***1–4*** show representative coronal brain slices (100 μm thick) labeled with NeuN antibody 1 week after surgery. The *ex vivo* analysis does not find regions of tissue suffering or necrosis due to craniotomy or laser irradiation. Scale bar, 1 mm. On the left, panels ***5–8*** show representative coronal brain slices (1 mm thick), 24 h after surgery and intravenous injection of Evans Blue dye. The absence of blue staining highlights that the surgery followed by green laser illumination does not induce BBB permeability alterations. Download Figure 1-1, TIF file.

Five minutes after the injection of the dye, a 532 nm green laser was focused before the MCA branch for 25 min to promote the formation of a stable clot and the consequent occlusion of the distal branch of the MCA. The green laser used for the experiments focused on the blood vessel and did not heat the irradiated tissue near the MCA during photoirradiation, as shown by the presence of perfused blood vessels near the illumination site. At the end of the procedure, the muscle over the bone will be replaced and the skin sutured. Mice were placed in their cages until full recovery.

### *Ex vivo* evaluation of blood–brain barrier permeability

To perform an *ex vivo* evaluation of blood–brain barrier permeability, we injected 0.20 ml of Evans Blue (EB) dye (0.20 mg/ml) into the mouse tail vein at the end of the surgery to occlude the distal branch of the MCA. Twenty-four hours after the injection, the animal was anesthetized by an intraperitoneal injection of ketamine (100 mg/kg) and xylazine (10 mg/kg) and then perfused with 100 ml of PBS to remove the blood from the brain tissue. The brain was then extracted and placed in paraformaldehyde (PFA) 4% for 1 h. Then the brain was sectioned with a brain matrix producing ∼10 slices that were 1 mm thick.

### Brain water content evaluation

The evaluation of brain water content was performed following the method previously applied by Kenne et al. (2012). Twenty-four hours after the occlusion of the MCA, mice were killed with an overdose of anesthetic. The brain was divided along the midline, and the contralateral and ipsilateral tissue was weighed right after removal to obtain wet weight (WW). The tissue was then dried at 60°C for 72 h and weighed to obtain dry weight (DW). Water content was calculated as follows: Water Content = (WW – DW)/(DW). Tissue swelling was calculated as a percentage of the ratio between the variation of the wet weight and the initial wet weight: [(Final WW – Initial WW)/(Initial WW)] * 100.

### Clasping test

**Figure 2.**
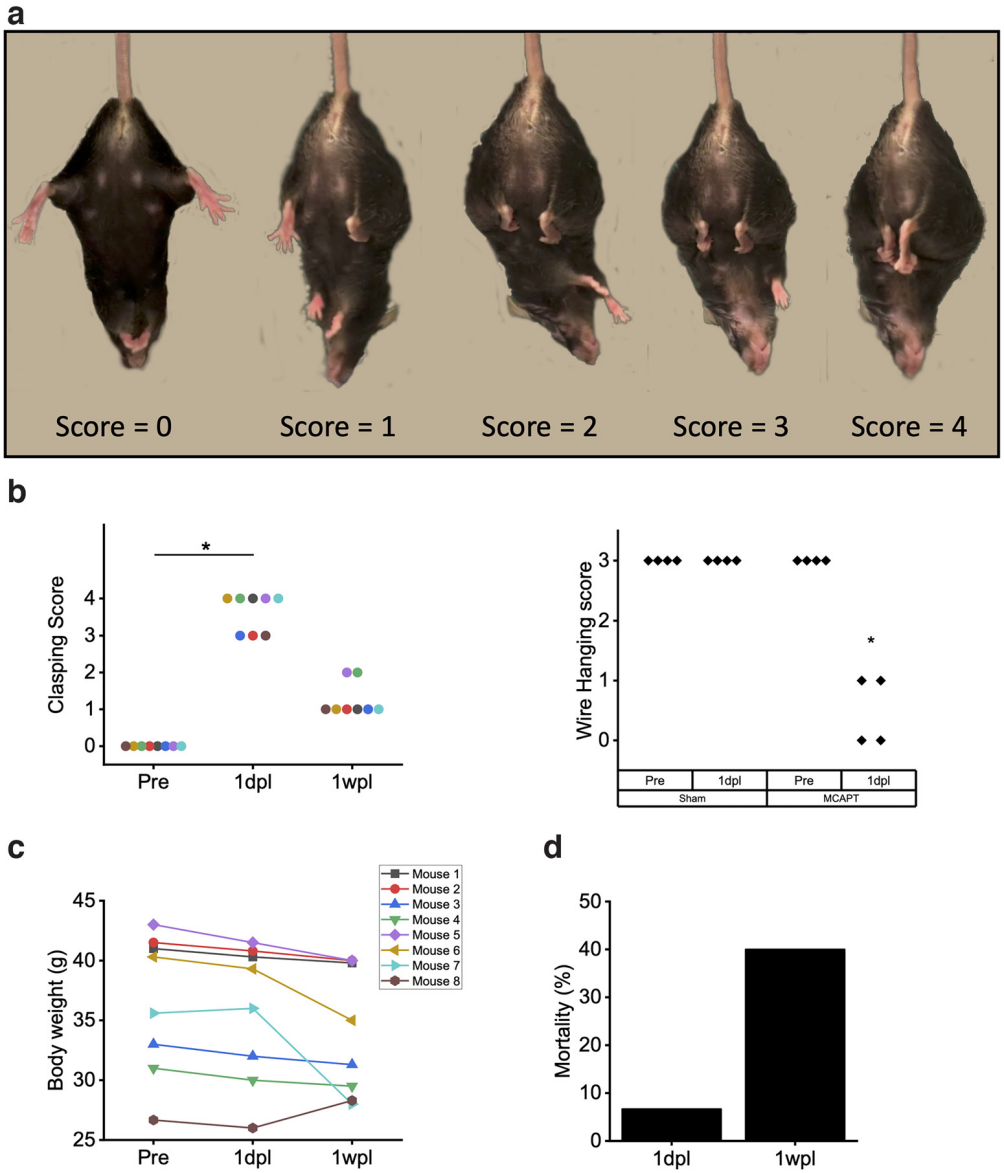
MCAPT induces severe dystonia in the acute phase after stroke: ***a***, Representative pictures of mice during the clasping test. A score of 0 was assigned to mice with no clasping reflex; 1, 2, 3, and 4 were assigned respectively when one, two, three, and four limbs are retracted on the abdomen. ***b***, Left, The clasping reflex revealed a tendency to higher clasping behavior after stroke both in the acute phase (at 1dpl) and at 1wpl. *p value based on one-way repeated-measures ANOVA followed by a *post hoc* Tukey’s HSD test: Pre-1dpl, *p* = 0; Pre-1wpl, *p* = 1.57E-05; 1dpl-1wpl, *p* = 0. Right, The wire-hanging test revealed a decrease in the strength of mice forelimbs 24 h after the damage. *p value based on one-way repeated-measures ANOVA followed by a *post hoc* Tukey’s HSD test: MCAPT Pre-1dpl, *p* = 1.83E-5; 1dpl Sham-MCAPT, *p* = 1.83E-5. ***c***, The graph shows the weight of mice measured at the three time points. ***d***, The graph shows the mortality rate 24 h and 1 week after the lesion (*n* = 15). See also Extended Data Figure 2-1.

The clasping behavior was induced by suspending the mouse from the base of the tail 10 cm above the cage for 20 s. We assigned a score of 0 for no clasp if the limbs are splayed outward away from the abdomen. If one limb is retracted toward the stomach for >50% of the time suspended, we assigned a score of 1. If two limbs are retracted toward the stomach for >50% of the time suspended, we assigned a score of 2. If three limbs are retracted toward the stomach for >50% of the time suspended, we assigned a score of 3. If both forelimbs and hindlimbs touch and press on the stomach, indicating a severe clasp, we assigned a score of 4 (Fig. 2*a*). At the end of the test, the animal was placed into its cage.

10.1523/ENEURO.0244-22.2022.f2-1 Figure 2-1 ***a***, Body weight evaluation of Sham group at three different time points Pre, 1dpl, and 1wpl, respectively. ***b***, As observed in MCAPT mice, the clasping reflex revealed a tendency to higher clasping behavior after stroke in the acute phase (1dpl) in the EB group as well as in the MCAPT group. *p value based on one-way repeated-measures ANOVA followed by *post hoc* Tukey’s correction: Pre-1dpl, *p* = 0.00002. ***c***, Body weight monitoring does not highlight any alteration after the MCA occlusion. Download Figure 2-1, TIF file.

### Wire-hanging test

To evaluate grip strength, balance, and endurance 24 h after the injury, we tested mice in the wire-hanging test (Balkaya et al., 2013). Mice were brought by the tail near a 2-mm-thick metallic wire maintained 35 cm above a layer of bedding material to prevent injury to the animal in case of falls. When the animal hung to the wire with the forelimb, the mouse was released by the operator. If the animal reached one end of the wire the score was increased by 1. If the animal fell, the score was diminished by 1 and the elapsed time was noted. Mice performed three trials to obtain the final score.

### Immunohistochemical analysis

For *ex vivo* investigation, stroke or sham-operated mice were transcardially perfused with 4% PFA on day 7 after surgery. Brains were cut using a vibrating-blade vibratome (Leica) to obtain 100-μm-thick coronal sections that were used for immunostaining of the neuronal marker NeuN (1:1000; anti-NeuN chicken, Millipore) and glial fibrillary acidic protein (GFAP; 1:1000; anti-GFAP rabbit, Abcam).

The NeuN immunostaining was performed to quantify the lesion volume 1 week after PT. The stroke volume for each animal was calculated by summing up all damaged areas and multiplying the number by section thicknesses and by the spacing factor 4 (Conti et al., 2022). Images were acquired with a (Stemi 508, Carl Zeiss). The total volume in cubic millimeters is given as the mean ± SE of all analyzed animals (*n* = 6). The experimenter was blind to the experimental group of the samples.

The number of GFAP-positive neurons was analyzed using a confocal fluorescence microscope (model Eclipse TE 300, Nikon) with a Nikon Plan EPO 60× objective (numerical aperture 1.4, oil-immersion, Nikon). We decided to focus our investigation on the following four regions of interest (ROIs): the peri-infarct area [ischemic border zone ipsilesional (IBZ_IL_)]; a region in the ipsilesional hemisphere distant to the stroke core [remote zone IL (RZ_IL_)]; a region contralateral (CL) to the peri-infarct area [ischemic border zone CL (IBZ_CL_)]; and a region in the healthy hemisphere CL to the ischemic core (IC_CL_). For each ROI (IBZ_IL_, RZ_IL_, IBZ_CL_, IC_CL_), we acquired three fields of view. The density of GFAP-positive cells was evaluated considering the following criteria: (1) the same brightness/contrast value was set for all images; (2) cells placed at the border of the image were not counted; (3) cells that were not clearly visible were excluded and therefore not counted; and (4) specific signals of the background were excluded.

The morphologic analysis of astrocytes was performed using two strategies: Sholl’s method and the Skeleton analysis. For each of the five animals, three slices of the brain, central to the damage, were analyzed. In each slice, we acquired three images (212.13 × 212.13 μm) for each ROI, and in each image, we identified three astrocytes. One hundred eight astrocytes per animal were analyzed. We used four animals for the analysis of the sham mice. For each animal, we analyzed three slices, and for each slice we analyzed three astrocytes for each of the four ROIs (IBZ_IL_, RZ_IL_, IBZ_CL_, IC_CL_). A total of 144 astrocytes was analyzed.

By applying Sholl’s method (ImageJ software), we isolated each individual astrocyte, and, starting from the soma, we drew concentric circles around it, at a distance of 3 μm from each other. This method allows quantifying the number of intersections of each astrocytic process with a single circumference and the total number of intersections.

We used Skeleton analysis (ImageJ software) to determine the number and length of primary processes, the number of junctions, the number of end points, the average length of the processes, and finally the maximum length of the branches of the astrocytes. Since in Sham mice we did not reveal any significant differences among the four ROIs, we considered a mean value for each parameter in the main figures of the manuscript and added the Sham analysis for each ROI (IBZ_IL_, RZ_IL_, IBZ_CL_, IC_CL_) in the Extended Data Figs. 4-2, 5-1, 5-2.

### Statistical analysis

All the analyses performed in both the *in vivo* and *ex vivo* experiments were performed blind. Moreover, all the data were independently evaluated by the two researchers that performed the experiments and the analysis. Results were considered statistically significant if their corresponding *p* value was ≤0.05. OriginPro software (OriginLab) was used for all other statistical analyses. For all ANOVAs that were statistically significant, multiple comparisons among time points and different regions of the cortex were assessed using repeated-measures ANOVA followed by a *post hoc* Tukey’s HSD test.

## Results

### A novel single-vessel photothrombotic stroke mouse model

We developed a novel method to permanently induce light-mediated occlusion of the distal branch of the MCA in mice (Fig. 1*a*,*b*). The MCA was exposed through a small craniotomy (Fig. 1*b*, left). Then, 5 min after the intraperitoneal injection of Rose Bengal, the MCA was illuminated for 25 min with a green laser (Fig. 1*b*, middle), which promoted the formation of a stable clot (Fig. 1*b*, right) and, consequently, blood perfusion interruption in the downstream brain tissue. We performed the MCAPT in two different experimental groups (Fig. 1*c*). In the first group (MCAPT) we performed behavioral experiments the day before the stroke (Pre) and then 24 hours (one day post lesion, 1dpl) and one week (one week post lesion, 1wpl) after the photothrombosis. After behavioral evaluations, mice were perfused to perform *ex vivo* experiments. In the second group (EB), mice were tested 1 d before and 1 d after stroke. At the end of photothrombosis, we injected Evans Blue serum albumin-binding dye into the mouse tail vein to determine the presence of extravasation (i.e., hemorrhage and edema) in brain tissue 1 d after damage. For both experimental groups (MCAPT and EB), we performed a set of experiment in which mice were subjected to the same surgery and procedure with respect to MCAPT and EB mice, respectively, except for the Rose Bengal injection, which was replaced by injection of the same volume of saline. At the end of the experimental period, mice were killed to performed *ex vivo* evaluations (Extended Data Fig. 1-1). To quantify the lesion volume induced by the photothrombotic occlusion of the MCA 1 week after stroke, the perfused brain was cut into 100 μm coronal sections. The NeuN immunostaining highlighted a region of dead tissue affecting only the mouse cortex extending from motor regions up to visual areas, in the rostrocaudal direction (Fig. 1*d*), with an overall lesion volume of 6.9 ± 0.1 mm^3^ in stroked mice (Fig. 1*e*). Sham mice did not show any sign of tissue suffering ascribable to craniotomy or laser irradiation (Extended Data Fig. 1-1, panels 1–4).

### MCAPT induces severe dystonia in poststroke acute phase

To assess the functional impairment caused by photothrombosis, we performed the clasping test (Guyenet et al., 2010; Miedel et al., 2017) at different time points (Fig. 2*a*). While in healthy conditions, all the mice splay the limbs outward, indicating the physiological reflex to grab something when hanged, 1 d after MCA photothrombosis we observed a considerable worsening of motor performance, only partially recovered 1 week after stroke (Fig. 2*b*). Then to better characterize motor performances in the acute phase after stroke, we tested a subgroup of mice in the wire-hanging test (MCAPT, *n* = 4; Sham, *n* = 4). While the final score remained unaltered 24 h after the surgery in Sham mice, we observed in MCAPT animals a severe worsening of the grip strength, balance, and endurance (Fig. 2*b*, right). Body weight evaluation did not highlight any significant difference before and after photothrombosis (Fig. 2*c*), though mouse body weight variations are higher in the MCAPT group compared with the Sham group (Extended Data Fig. 2-1*a*). The permanent occlusion of the distal branch of the MCA is lethal for 6.6% (*n* = 1) of mice 1 d after irradiation, and 40% (*n* = 5) 1 week after stroke (Fig. 2*d*).

### MCAPT induces blood–brain barrier leakage and edema formation in the ipsilesional hemisphere

**Figure 3.**
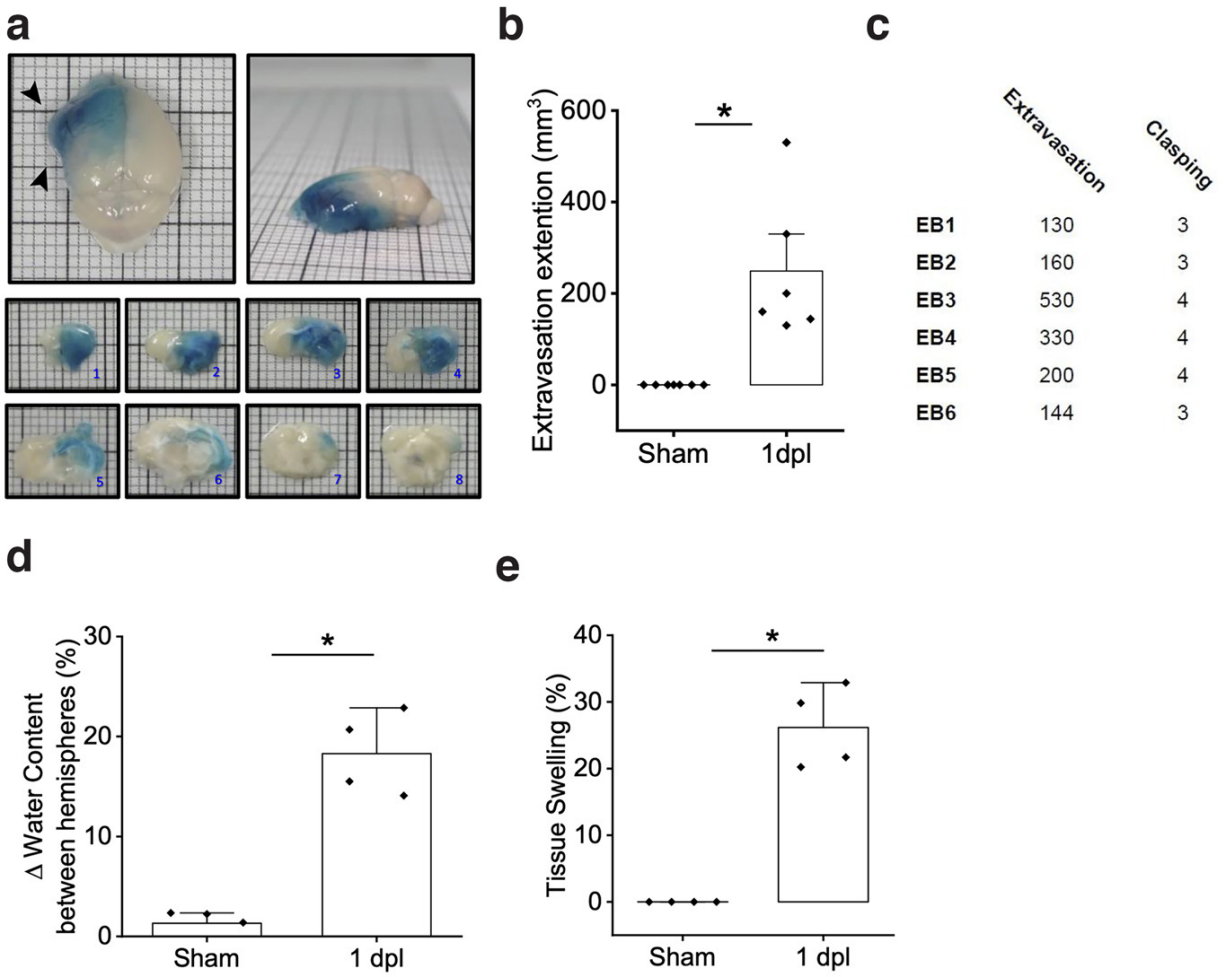
MCAPT induces blood–brain barrier leakage and edema formation in the ipsilesional hemisphere. ***a***, Dorsal (left) and lateral (right) pictures of a representative MCAPT brain of a mouse injected in the tail vein with Evans Blue dye right after photothrombosis. ***1–8***, Coronal sections of the same animal. Black arrows point to tissue swelling. ***b***, *Ex vivo* quantification of the brain tissue presenting a blue signal in the Sham group and the EB group (1dpl) 1 d after the photothrombotic occlusion. *p = 0.003 based on one-way ANOVA followed by a *post hoc* Tukey’s HSD test (*n* = 6). ***c***, The table shows the clasping test score and the extension of extravasation for each EB mouse. ***d***, Brain water content evaluation 24 h after damage highlights the increase of wet weight in the ipsilesional hemisphere of MCAPT mice with respect to Sham mice. *p = 0.003 based on one-way ANOVA followed by a *post hoc* Tukey’s HSD test (*n* = 4). ***e***, Tissue swelling evaluation 24 h after stroke shows the emergence of brain tissue distortion affecting the ipsilesional hemisphere of MCAPT mice. *p = 0.0001 based on one-way ANOVA followed by a *post hoc* Tukey’s HSD test (*n* = 4).

We then wondered whether the high percentage of mortality observed during the first week after the MCA occlusion (Fig. 2*d*) was caused by the emergence of blood–brain barrier alterations (i.e., hemorrhage and edema). To clarify this aspect, we used Evans Blue, an organic dye characterized by a very high affinity for serum albumin, which allows a rapid and low-cost assessment of BBB permeability (Saunders et al., 2015). The loss of blood–brain barrier integrity and the consequent extravasation were evaluated by quantifying the presence of blue staining of cerebral tissue, caused by leakage of the dye from the blood vessels to the brain parenchyma (Stoll et al., 2009; Yang et al., 2017). Therefore, in EB mice (*n* = 6), we injected the Evans Blue dye into the mouse tail vein right after photothrombosis. Mice were then killed 24 h after the injection. While in Sham mice (*n* = 4) no evidence of blood–brain barrier alterations were revealed (Extended Data Fig. 1-1, panels 5–8), in MCAPT mice we observed that the diffusion of the dye affects a large portion of the ipsilesional hemisphere, extending both in the rostral direction up to the olfactory bulbs and in the caudal regions, (Fig. 3*a*,*b*). Moreover, the tissue appears to be swollen around the stroke core (Fig. 3*a*, black arrows). These animals, evaluated through the clasping test before (Pre) and 1 day after stroke (1dpl), showed behavioral deficits comparable to the MCAPT group and no consistent alterations in body weight (Extended Data Fig. 2-1*b*,*c*). In particular, mice with more severe impairment are characterized by a higher extension of extravasation (Fig. 3*c*). Since this preliminary evaluation suggested the presence of a barrier leak, to better quantify the presence of edema 24 h after the photothrombotic damage, we evaluated the brain water content of ipsilesional and contralesional hemispheres as a measure of cerebral edema (Kenne et al., 2012) in another group of mice (Sham, *n* = 4; MCAPT, *n* = 4). The comparison of the variation of water content between hemispheres of Sham with respect to MCAPT mice showed a significant increase in water after the photothrombotic occlusion of the MCA (Fig. 3*d*). Moreover, a significant increment of tissue swelling was observed in the MCAPT group, which was not found in Sham mice (Fig. 3*e*).

### MCAPT increases astrocytes density and complexity in the peri-infarct cortex

**Figure 4.**
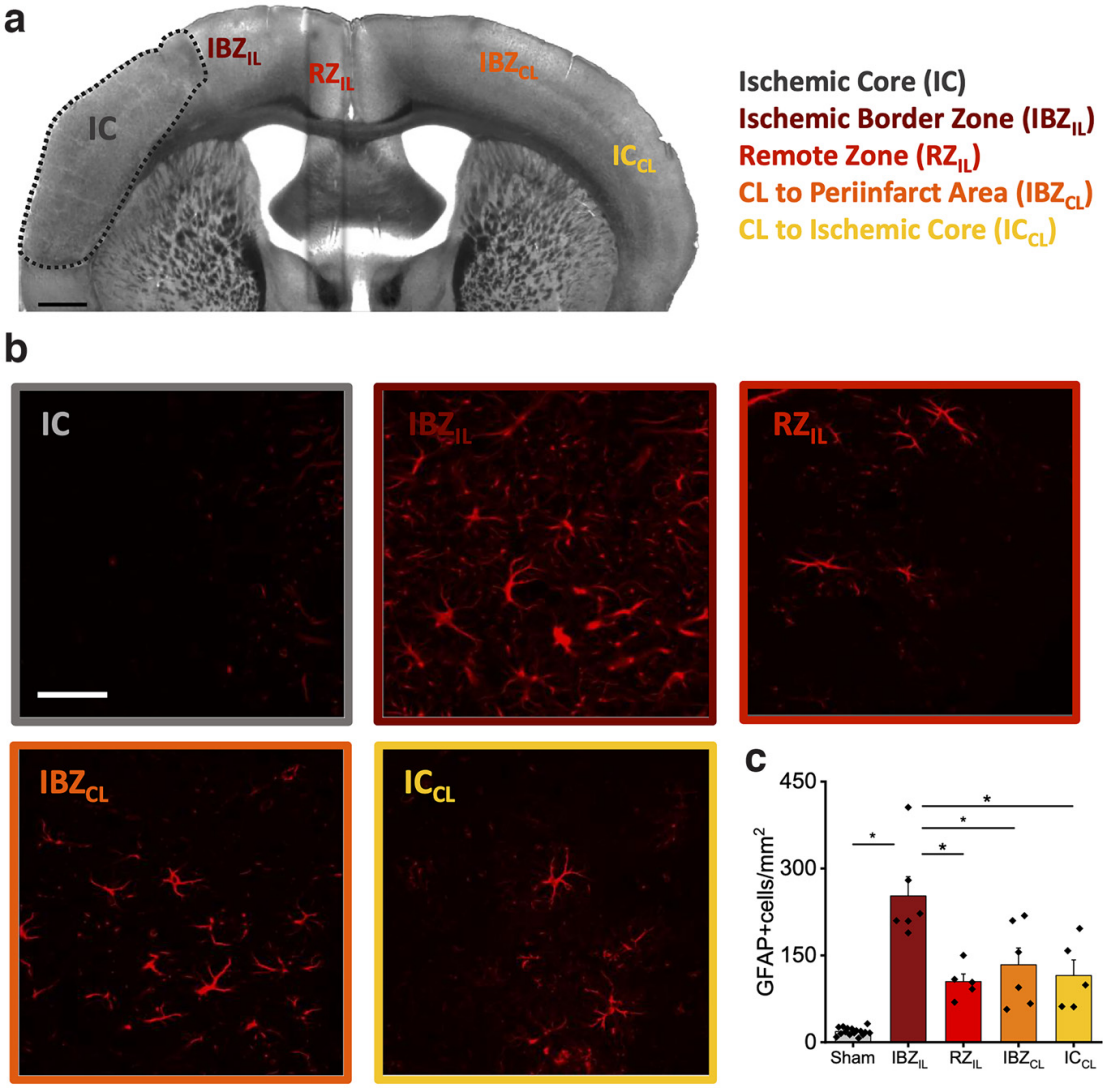
MCAPT increases astrocyte density in the peri-infarct area. ***a***, A representative brain slice highlighted the IC and the 4 ROIs identified for the astrocytes analysis. Scale bar, 0.5 mm. ***b***, Representative field of view of each ROI acquired with a confocal microscope. Scale bar, 45 μm. ***c***, The graph shows the density (average ± SEM) of GFAP-positive cells in the 4 ROIs (IBZ_IL_= 252.57 ± 33.07; RZ_IL_ = 104.63 ± 13.23; IBZ_CL_ = 133.64 ± 29.11; IC_CL_ = 115.18 ± 26.894). *p* value based on one-way ANOVA followed by a *post hoc* Tukey’s HSD test. See Extended Data Table 4-1: IBZ_IL_-RZ_IL_, *p* = 0.00,721; IBZ_IL_-IBZ_CL_, *p* = 0.02,508; IBZ_IL_-IC_CL_, *p* = 0.01278. See also Extended Data Figures 4-1 and 4-2.

**Figure 5.**
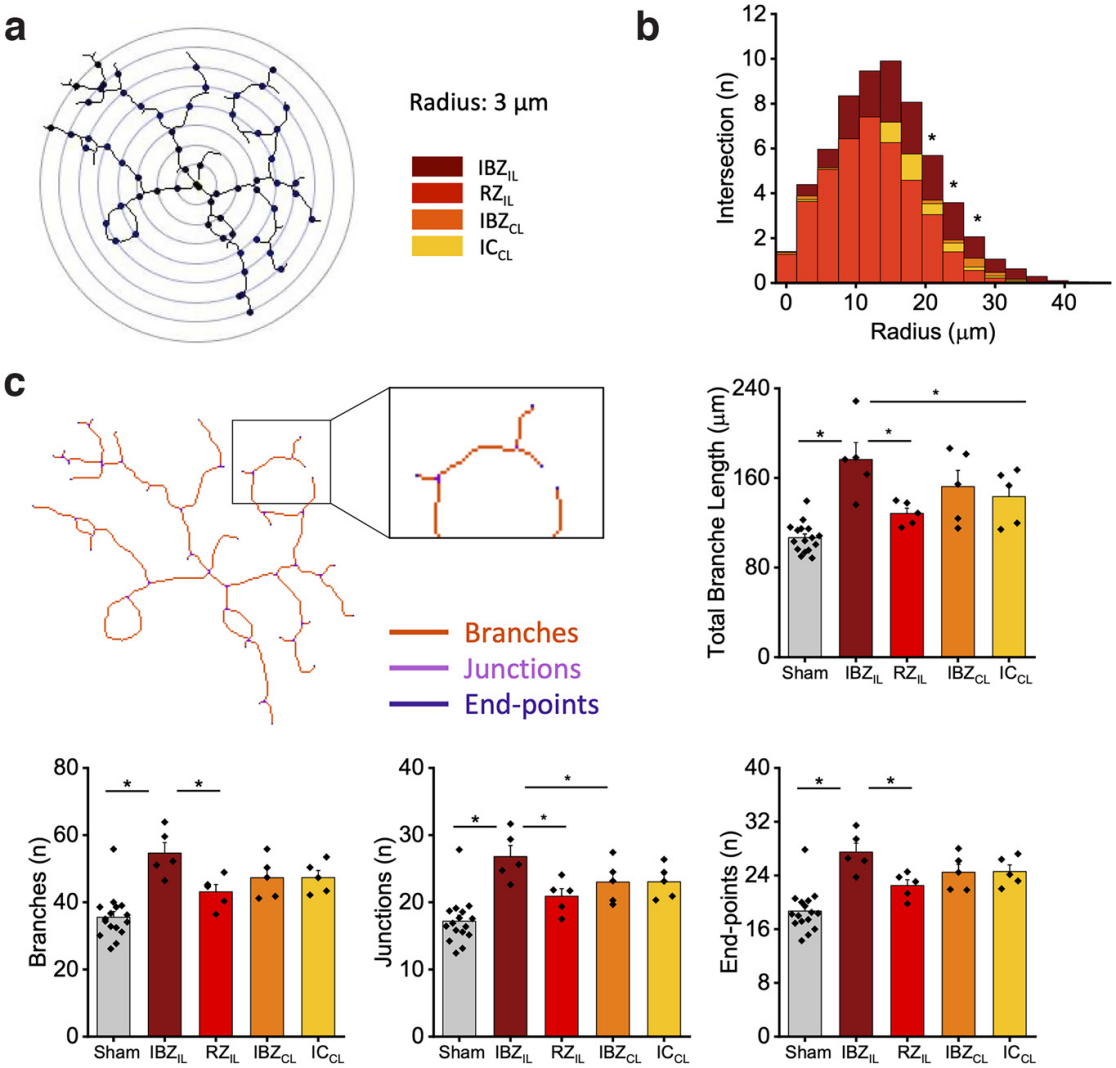
MCAPT increases astrocyte complexity in the peri-infarct area of MCAPT mice. ***a***, Representative image of an astrocyte analyzed with the Sholl method. ***b***, The graph shows the distribution of the number of intersections for each radius in the 4 ROIs. *p* value based on two-way repeated-measures ANOVA followed by a *post hoc* Tukey’s HSD test. See Extended Data Tables 5-1, 5-2, and 5-3: radius 7: IBZ_IL_-RZ_I_, *p* = 2.51E-08; IBZ_IL_-IBZ_CL_, *p* = 0.006; IBZ_IL_-IC_CL_, *p* = 0.006; radius 8: IBZ_IL_-RZ_IL_, *p* = 8.65E-07; IBZ_IL_-IBZ_CL_, *p* = 0.013; IBZ_IL_-IC_CL_, *p* = 1.72E-04; radius 9: IBZ_IL_-RZ_IL_, *p* = 6.82E-06; IBZ_IL_-IBZ_CL_, *p* = 0.029; IBZ_IL_-IC_CL_, *p* = 4.10E-04. ***c***, Representative image of the same astrocyte in ***a*** analyzed with the Skeleton analysis. All the features of astrocytes in the 4 ROIs are shown as the average ± SEM. The intergroup statistical analysis was performed through a two-way repeated-measures ANOVA followed by a *post hoc* Tukey’s HSD test; see Extended Data Table 5-4. The intragroup statistical analysis was performed through a one-way repeated-measures ANOVA followed by a *post hoc* Tukey’s HSD test; see Extended Data Tables 5-5 and 5-6. Total branches length sham = 106.71 ± 3.32; IBZ_IL_= 176.53 ± 15.04; RZ_IL_= 128.32 ± 4.74; IBZ_CL_ = 152.23 ± 14.49; IC_CL_ = 143.28 ± 11.04; intergroup analysis: IBZ_IL_ MCAPT-Sham, *p* = 0.002; intragroup analysis: IBZ_IL_-RZ_IL_, *p* = 0.003; IBZ_IL_-IC_CL_, *p* = 0.033. Number of astrocytes, branches: Sham = 35.54 ± 1.68; IBZ_IL_ = 54.645 ± 3.126; RZ_IL_ = 43.127 ± 2.161; IBZ_CL_ = 47.307 ± 2.742; IC_CL_ = 47.341 ± 2.113; intergroup analysis: IBZ_IL_ MCAPT-Sham, *p* = 0.001; intragroup analysis: IBZ_IL_-RZ_IL_, *p* = 0.044. Number of astrocytes, junctions: Sham = 17.18 ± 0.87; IBZ_IL_ = 26.81 ± 1.63; RZ_IL_ = 20.89 ± 1.13; IBZ_CL_ = 23.01 ± 1.421 IC_CL_ = 23.04 ± 1.12; intergroup analysis: IBZ_IL_ MCAPT-Sham, *p* = 0.03; intragroup analysis: IBZ_IL_-RZ_IL_, *p* = 0.026; IBZ_IL_-IC_CL_, *p* = 0.042. Number of astrocytes, end points: Sham = 18.68 ± 0.77; IBZ_IL_ = 27.47 ± 1.34; RZ_IL_ = 22.49 ± 0.86; IBZ_CL_ = 24.47 ± 1.20; IC_CL_ = 24.58 ± 0.97; intergroup analysis: IBZ_IL_ MCAPT-Sham, *p* = 0.02; intragroup analysis: IBZ_IL_-RZ_IL_, *p* = 0.044. See also Extended Data Figures 5-1 and 5-2.

To quantify the inflammation induced by the damage, we analyzed astrocytes in different regions of the brain in MCAPT and Sham mice. We identified the following four regions of interest (Fig. 4*a*) within the cortex: ipsilesional ischemic border zone (IBZ_IL_), remote zone (RZ_IL_), contralesional ischemic border zone (IBZ_CL_), contralesional ischemic core (IC_CL_), Extended Data Fig. 4-1. In the ischemic core (IC), no fluorescence signal was revealed. At a glance, as shown in Figure 4*b*, the IBZ_IL_ in MCAPT animals was characterized by an intense fluorescence signal compared with other regions. The analysis revealed an increase of GFAP-positive astrocytes in the peri-infarct area (IBZ_IL_) of MCAPT animals with respect to Sham mice (Extended Data Fig. 4-2). Moreover, astrocyte density in the IBZ_IL_ of MCAPT mice was significantly higher with respect to other regions both in the ipsilesional and contralesional cortices (Fig. 4*c*, Extended Data Table 4-1). Then, by quantifying the number of branch intersections through Sholl analysis (Fig. 5*a*), we observed a significant increment of the intersection number (21–27 μm from the cell body) of IBZ_IL_ astrocytes compared with other regions of MCAPT mice (Extended Data Table 5-1). Conversely, in Sham animals, no differences were revealed between the ROIs at increasing distances from the cell body (Extended Data Fig. 5-1, Extended Data Table 5-2, Table 5-3). Finally, to further investigate astrocyte morphology, we performed Skeleton analysis to quantify the length of astrocytic processes as well as the number of branches, junctions, and end points (Fig. 5*c*, Extended Data Fig. 5-2). Astrocyte morphology is consistent among all the ROIs in Sham mice (Extended Data Fig. 5-2*b*). In detail, astrocytes show a lower number of branches, junctions, and end points in all the analyzed regions compared with MCAPT mice (Fig. 5*c*, Extended Data Fig. 5-2*b*, Extended Data Tables 5-4, 5-5). Conversely, MCAPT mice showed strong differences in morphologic features between the regions observed (Fig. 5*c*, Extended Data Table 5-6). In particular, in the ipsilesional hemisphere the analysis revealed a significant difference in the number of branches, junctions, and end points between IBZ_IL_ and RZ_IL_. Finally, in the contralesional hemisphere, all morphologic parameters were comparable between the two regions analyzed.

10.1523/ENEURO.0244-22.2022.f4-1 Figure 4-1 GFAP analysis. Representative images of GFAP-labeled astrocytes in the four different regions of interest (IBZ_IL_, RZ_IL_, IBZ_CL_, IC_CL_) for each mouse. Download Figure 4-1, TIF file.

10.1523/ENEURO.0244-22.2022.f4-2 Figure 4-2 Astrocytes density in Sham mice: on the left, a representative image of anti-GFAP-labeled astrocytes. Scale bar, 45 μm. The graph on the right shows the density (average ± SEM) of GFAP-positive cells in the 4 ROIs (IBZ_IL_ = 18 ± 2.2; RZ_IL_ = 17 ± 3.6; IBZ_CL_ = 18 ± 3.1; IC_CL_ = 20.7 ± 5.1). Download Figure 4-2, TIF file.

10.1523/ENEURO.0244-22.2022.tab4-1 Table 4-1 Astrocytes density intragroup (Sham and MCAPT) and intergroups comparison. One-way repeated-measures ANOVA followed by Tukey’s test was used for intragroup comparison. Two-way repeated-measures ANOVA followed by Tukey’s test was used for intergroup comparison. Colored cells indicate *p*-values < 0.05. Download Table 4-1, DOC file.

10.1523/ENEURO.0244-22.2022.f5-1 Figure 5-1 Sholl analysis in MCAPT and Sham mice. The graphs show the distribution of the number of intersections for each radius in the 4 ROIs, color coded as in Figure 5. Download Figure 5-1, TIF file.

10.1523/ENEURO.0244-22.2022.f5-2 Figure 5-2 ***a***, ***b***, Skeleton analysis of astrocytes in MCAPT mice (***a***) and Sham (***b***) mice. All the parameters evaluated in the 4 ROIs are shown as the average ± SEM. The intergroup statistical analysis was performed through a two-way repeated-measures ANOVA followed by a *post hoc* Tukey’s HSD test (see Extended Data Table 5-4). The intragroup statistical analysis was performed through a one-way repeated-measures ANOVA followed by a *post hoc* Tukey’s HSD test (see Extended Data Tables 5-5, Tables 5-6). ***a***, Junctions (pixel): Sham = 56.37 ± 3.37; IBZ_IL_ = 85.74 ± 4.93; RZ_IL_ = 68.45 ± 3.5; IBZ_CL_ = 74.01 ± 4.36 IC_CL_ = 75.47 ± 4.2; intergroup analysis: IBZ_IL_ MCAPT-Sham, *p* = 0.00009. Average length of branches (μm): Sham = 4.28 ± 0.14; IBZ_IL_ = 4.35 ± 0.18; RZ_IL_ = 3.81 ± 0.24; IBZ_CL_ = 4.38 ± 0.18; IC_CL_ = 3.96 ± 0.23; intergroup analysis: IBZ_IL_ MCAPT-Sham, *p* = 0.03; intragroup analysis: IBZ_IL_-RZ_IL_, *p* = 0.01; IBZ_CL_-RZ_IL_, *p* = 0.007; IBZ_CL_-IC_CL_, *p* = 0.05. Maximum length of branches (μm): Sham = 14.01 ± 0.37; IBZ_IL_ = 16.11 ± 0.42; RZ_IL_ = 13.95 ± 1.04; IBZ_CL_ = 15.6 ± 1.37; IC_CL_ = 13.53 ± 0.87. ***b***, Total length of branches (μm): Sham, IBZ_IL_ = 112.914 ± 9.819; RZ_IL_ = 106.157 ± 5.648; IBZ_CL_ = 104.43 ± 6.303; IC_CL_ = 193.352 ± 5.869. Number of astrocytes in branches (average ± SEM) in the 4 ROIs: IBZ_IL_ = 40.87 ± 5.15; RZ_IL_ = 34.49 ± 1.7 IBZ_CL_ = 34.05 ± 2.51; IC_CL_ = 32.76 ± 2.84. Number of astrocytes in junctions: IBZ_IL_ = 19.91 ± 2.71; RZ_IL_ = 16.74 ± 0.87; IBZ_CL_ = 16.35 ± 1.29; IC_CL_ = 15.73 ± 1.45. Number of astrocytes at end points in the 4 ROIs: IBZ_IL_ = 21.23 ± 2.28; RZ_IL_ = 17.79 ± 0.84; IBZ_CL_ = 18.19 ± 1.18; IC_CL_ = 17.519 ± 1.317. Junctions (pixels): IBZ_IL_ = 67.39 ± 11.48; RZ_IL_ = 54.63 ± 2.02; IBZ_CL_ = 52.56 ± 3.55; IC_CL_ = 50.88 ± 4.79. Average length of branches (μm): IBZ_IL_ = 3.9 ± 0.15; RZ_IL_ = 4.3 ± 0.25; IBZ_CL_ = 4.37 ± 0.21; IC_CL_ = 4.54 ± 0.47. Maximum length of branches (μm): IBZ_IL_ = 13.90 ± 0.27; RZ_IL_ = 14.15 ± 0.62; IBZ_CL_ = 14 ± 0.51; IC_CL_ = 14 ± 1.44. Download Figure 5-2, TIF file.

10.1523/ENEURO.0244-22.2022.tab5-1 Table 5-1 Intragroup (MCAPT) comparison of Sholl analysis in different regions of the cortex. Two-way repeated-measures ANOVA followed by Tukey’s test. Colored cells indicate *p*-values < 0.05. Download Table 5-1, DOC file.

10.1523/ENEURO.0244-22.2022.tab5-2 Table 5-2 Intragroup (Sham) comparison of Sholl analysis in different regions of the cortex. Two-way repeated-measures ANOVA followed by Tukey’s test. Colored cells indicate *p*-values < 0.05. Download Table 5-2, DOC file.

10.1523/ENEURO.0244-22.2022.tab5-3 Table 5-3 Intergroup (MCAPT and Sham) comparison of Sholl analysis for each region of the cortex. Two-way repeated-measures ANOVA followed by Tukey’s test. Colored cells indicate *p*-values < 0.05. Download Table 5-3, DOC file.

10.1523/ENEURO.0244-22.2022.tab5-4 Table 5-4 Intergroup (MCAPT and Sham) comparison of Skeleton analysis for each region of the cortex. Two-way repeated-measures ANOVA followed by Tukey’s test. Colored cells indicate *p*-values < 0.05. Download Table 5-4, DOC file.

10.1523/ENEURO.0244-22.2022.tab5-5 Table 5-5 Intragroup (Sham) comparison of Skeleton analysis in different regions of the cortex. One-way repeated-measures ANOVA followed by Tukey’s test. Colored cells indicate *p*-values < 0.05. Download Table 5-5, DOC file.

10.1523/ENEURO.0244-22.2022.tab5-6 Table 5-6 Intragroup (MCAPT) comparison of Skeleton analysis in different regions of the cortex. One-way repeated-measures ANOVA followed by Tukey’s test. Colored cells indicate *p*-values < 0.05. Download Table 5-6, DOC file.

This aspect highlights the establishment of an inflammatory regime in the acute phase after stroke involving both hemispheres, though especially prominent in the periinfarct cortex of MCAPT mice.

## Discussion

In this study, we adopted the photothrombotic technique to induce an ischemic occlusion of the distal branch of the middle cerebral artery in mice. Although a similar approach was successful in rats (Watson et al., 1987) and rabbits (Zhao et al., 2002), and in a “tandem occlusion” through the ligation of the common carotid artery in mice (Sugimori et al., 2004), to the best of our knowledge, this is the first study showing its application in mice.

The occlusion of the MCA can be achieved with other strategies such as the intraluminal insertion of a filament, the endothelin-1 model, and the ligation or cauterization of the blood vessel, resulting in different postinjury complications (Gonzalez and Kolb, 2003). As previously discussed, the intraluminal suture of the MCA technique is a widely used animal model of stroke. However, the insertion of the filament leads to obstruction of the hypothalamic artery, thus inducing hyperthermia and a consequent increase of infarct volume, worsening functional outcome (Zhao et al., 1994; Reglodi et al., 2000). Moreover, this model shows high variability of the infarct size resulting in low reproducibility and an unsatisfactory success rate of occlusion (Howells et al., 2010). Furthermore, the surgery to access and manipulate the vasculature requires skilled and experienced hands (Howells et al., 2010). The endothelin-1 technique is another stroke model commonly used both in rats and mice, based on the local application of a vasoconstrictor agent. The procedure is easy to perform and allows the control of vessel vasoconstriction modulating the dose of the vasoconstrictive agent. However, this approach is characterized by high variability in stroke volume (Braeuninger and Kleinschnitz, 2009). Similarly, the cauterization of the MCA is characterized by low reproducibility (Mora-Lee et al., 2011) and presents several drawbacks, including possible damage to the dura mater and tissue surrounding the vessel. Furthermore, cauterization induces permanent damage, not amenable to reperfusion by removing the suture filament, or by light-induced recanalization thrombolytic agents (Ishrat et al., 2009). Conversely, the photothrombotic model has the advantage of inducing the formation of platelet-rich and fibrin-rich thrombi in the blood vessels of the irradiated site (Matsuno et al., 1993; Saniabadi et al., 1995). This approach is minimally invasive and is capable of inducing highly reproducible cortical damage both in rats and mice, targeting with high precision the location of ischemia (Macrae, 2011). Moreover, photothrombosis has the great advantage of tuning the plasma concentration of the dye, and the intensity and duration of the light to control the size and the depth of the lesion. Depending on the procedure applied, the target of photothrombosis ranges from an extended region of the cortex to a single capillary.

In previous studies, the photothrombotic approach was applied to single blood vessels within the mouse brain cortex (Shih et al., 2013). This strategy has on the one hand the advantage of being able to target the region of the damage to investigate the microscopic basis of cerebral ischemia, selecting a specific class of blood vessel (i.e., capillary or surface arteriole or venule) in a specific cortical area. By combining the stroke model with imaging setups equipped with multiple light sources, alterations of the vasculature (Clark et al., 2019; Sunil et al., 2020), and brain dynamics, such as cortical depolarizing waves, (Balbi et al., 2017) were monitored *in vivo*.

Here, by characterizing the photothrombotic occlusion of the distal branch of the MCA in mice, we observed the formation of a stable clot in the blood vessel after 25 min of laser irradiation that leads to reproducible extended damage in the mouse cortex 1 week after the lesion. Compared with both single-capillary and cortex-targeted photothrombosis, our model, targeting the distal branch of the MCA, induces a more severe lesion within the mouse brain cortex. Moreover, with respect to the cortical irradiation model, in which the distribution of pial microvasculature can vary between animals of different ages or strains (Labat-gest and Tomasi, 2013), the photothrombotic occlusion of the distal branch of the MCA enables high reproducibility. Furthermore, the model induces a strong behavioral deficit revealed by the clasping and wire-hanging tests, mimicking a severe human infarction. Moreover, the MCAPT induces a pronounced leakage of the BBB and edema formation, making it a suitable model to investigate the main consequences affecting human stroke patients (i.e., hemorrhagic transformation and cerebral edema). Indeed, during the acute phase postinjury, the strong behavioral impairment is accompanied by the alteration of the BBB. Previous studies using MCA occlusion to induce a cerebral stroke observed an increase in BBB permeability in the acute phase after the damage (Belayev et al., 1996; Rosenberg et al., 1998; Candelario-Jalil et al., 2022). In particular, Fernandez-Lopez et al. (2012) observed a marked increase in Evans Blue leakage in the injured cortex and in the caudate of adult rats. Moreover, many studies apply magnetic resonance imaging to noninvasively detect BBB leakage and edema after stroke injury (Knight et al., 2008; Taheri et al., 2009; Matsushita et al., 2013) after middle cerebral artery occlusion.

Finally, a strong upregulation of the GFAP was induced, indicating the activation of reactive astrogliosis during the acute phase (Li et al., 2014; Alia et al., 2021). Specifically, the increased density of GFAP-positive cells observed in the peri-infarct cortex of MCAPT mice 1 week after photothrombosis suggests the beginning of scar formation, as previously observed in other studies (Takamatsu et al., 2002; Shen et al., 2012).

Overall, the advantages of photothrombotic occlusion of the MCA include the possibility of producing large and consistent infarcts of the cortex by occluding a large blood vessel through a nonmechanical approach, maintaining the dura mater intact, and the intracranial pressure constant. Although this model requires a craniotomy, one of the main advantages of this method includes the relatively slight invasiveness and the high degree of reproducibility (Yao et al., 2003). Considering the extended edema and the high mortality rate observed, we deem the damage induced by the photothrombotic occlusion of the distal branch of the MCA to be severe. Indeed, our model aims at reproducing a severe stroke to study the acute consequences caused by large-vessel occlusion, and the high mortality observed is attributable to the high reproducibility of the model. Conversely, in human patients, the pathophysiological insurgence of ischemia is characterized by higher variability both in terms of occlusion site and comorbidities, thus resulting in a wider spectrum of patient outcomes. However, the photothrombotic model may allow controlling of the severity of the injury by tuning the irradiation time and the dye concentration (Macrae, 2011). Moreover, our model can also be applied to induce ischemia in neonatal mice. As previously assessed by Maxwell and Dyck (2005) the photothrombotic stroke model has several advantages with respect to conventional methods to induce damage in neonatal mice, including transient and permanent MCA occlusion. Indeed, the surgical difficulties of these approaches, due to MCA exposure and filament insertion are exacerbated when working with neonatal mice. Conversely, thanks to the transparency of pup skulls (Jia et al., 2018), the distal branch of the MCA is clearly visible, thus avoiding bone thinning or craniotomy for laser irradiation.

Since half of all ischemic strokes occur in MCA territory, the development of a reproducible mouse model of stroke mimicking large thromboembolic stroke in humans is crucial in preclinical animal studies. Moreover, an animal stroke model that better resembles the pathophysiology of human ischemic stroke allows the generation of preclinical datasets suitable for investigating network dynamics and functional biomarkers of poststroke recovery (Adam et al., 2020; Mascaro et al., 2020; Cecchini et al., 2021; Kreuz et al., 2022; Scaglione et al., 2022). Finally, this approach that, to our knowledge, has never been applied in mice, will allow vascular recanalization in future experiments by illuminating the occluded vessel with a specific wavelength, as previously demonstrated in rats (Watson et al., 2002; Yao et al., 2003). The light-induced recanalization will foster the investigation of the neurovascular mechanisms underneath the ischemic progression and will allow testing of neuroprotectant agents such as glyburide (Sheth et al., 2016, 2018). Indeed, previous clinical trials have shown that glyburide reduces brain swelling after ischemia, thus improving patients’ survival (Sheth et al., 2014; Simard et al., 2014). In particular, this neuroprotective agent has been proven to be effective in large hemispheric strokes at risk of cerebral edema. Indeed, since our novel stroke model induces alteration of BBB permeability and consequent brain edema, we believe that glyburide might be an appropriate pharmacological agent to be tested. The photothrombotic occlusion of the distal MCA model was developed thanks to a bidirectional collaborative approach between preclinical and clinical researchers [i.e., the TREES (Translational Research on Stroke) Study group]. The close collaboration between clinics and research is essential to facilitate the translation of mechanistic insights offered by animal models to the bedside and to build meaningful experimental studies based on real clinical needs (Conti et al., 2021).

## Synthesis

Reviewing Editor: Francisca Bronfman, Universidad Andres Bello Facultad de Medicina Decisions are customarily a result of the Reviewing Editor and the peer reviewers coming together and discussing their recommendations until a consensus is reached. When revisions are invited, a fact-based synthesis statement explaining their decision and outlining what is needed to prepare a revision will be listed below. The following reviewer(s) agreed to reveal their identity: Aditya Singh.

This paper describes a newly developed and characterized model of MCA stroke in elderly mice using a photothrombotic model. Several parameters were evaluated, including in vivo evaluations of mice behavior, body weight at 24 hours and seven days after stroke induction, the extension of the lesion through ex vivo immunostaining, blood-brain barrier (BBB) permeability 24 hours after stroke, and alterations of astrocytes morphology through ex vivo immunohistochemistry seven days after photothrombosis. Overall, the results reported are convincing, and the findings are well-written and presented. However, reviewers consider that the study needs additional revision and data. Please see below,

1-Results, please clarify

A-How has confirmed the blood perfusion interruption in downstream brain tissue?

B-How was the stability of occlusion after photothrombosis accessed for mice?

C-Since mortality could be due to other factors s.a. Evans blue, the statement related to mortality should indicate consideration for other factors and not only emphasize photothrombosis as a cause of death.

D-Are similar BBB permeability and edema effects observed for other modes of inducing a stroke? Provide references. If not, could such effects for photothrombosis arise from either heat-induced damage from the laser or Evans blue-related artifacts (refer to Saunders et al. 2015 Front. neurosci.) in this study?

2-Figure, please comment, correct, or add new data

A-Figure 1c - Mark the peri-infarct cortex and stroke the core with arrows.

Figure 1d - Add individual data points in the bar plot

C-Figure 2a, b - The difference between scores 1-5 is unclear from the pictures. Since the clasping score doesn’t fully reflect the behavioral effects of stroke and capture the variability in mice behavior, other behavioral measures of photothrombotic stroke (PS) should be provided or specify the limitation of the analysis performed.

D-Figure 2c - The body weight of sham animals should be provided to compare weight variations due to PS. If the mortality was up to 40%, it seems plausible that the severity of a stroke would be reflected in higher variations in body weight compared to sham animals.

E-Figure 2d - How many mice were used for the experiment before measuring the mortality rate? How many mice died? Add in methods.

F-Figure 3a - Was the tissue swelling, edema, and BBB leakage due to craniotomy, Evans blue, or PS? Considering the concerns raised by Saunders et al., 2015, Front. Neurosci. Against using EB to assess BBB leakage, additional experiments may be conducted with alternative markers for BBB leakage and edema formation, s.a., low and high molecular weight dextrans, etc. Alternatively, the supplementary data should provide EB-stained pictures for all the animals.

G-Figure 3b - Provide individual data points for the bar plots.

H-Related to Figure 3-1a,b - clasping behavior measure is insufficient evidence to claim EB does not affect behavior. Considering behavior is a broad term, statements about EB and behavior should be clear about which specific behavior is affected throughout the manuscript. Moreover, the absence of other behavioral measures such as open field, novel object recognition, etc., makes it difficult to justify the statements that using EB in the experiments doesn’t affect behavior. Finally, previous studies (Saunders et al. 2015) have discussed the potential lethal toxicity of EB, so the mortality rate observed in the study cannot solely be attributed to PS.

Overall, authors should provide additional evidence or back their claims with additional references for using EB. It would be best to compare the BBB leakage estimation with other markers.

I-Figure 4b - similar astrocyte images should be provided from each experimental animal,

J-Figure 4c - add individual data points to the bar plot. It should be clarified whether sham data includes average astrocyte numbers from all the subregions corresponding to IBZ, RZ, IC, or any other brain region.

K-Figure 5b - The color palette is not clear. More contrasting colors may be used for different subregions.

L-Figure 5 - all bar plots need individual data points

M-Figure 5-2a - Average branch length looks the same for sham and IBZ ipsilateral. What does the star mean for sham vs. IBZ-IL comparison? Does it match the information provided in the corresponding table? Individual data points are needed for all bar plots.

3-Methods, specify,

A-Optical layout of the laser-set up w.r.t mouse/brain orientation for photothrombosis should be added as a figure. All the information about the opto-mechanical components used for inducing PS should be added as a subsection in the methods.

B-What intensity of laser was used for photothrombosis (laser intensity at the focus)? Could there be damage in the tissue due to heating caused due to laser exposure during 25 min exposure?

C-Please consider that craniotomy could introduce potential artifacts due to tissue/blood vessel damage during surgery or post-surgery tissue swelling.

D-Related to Evans blue concentration. Two hundred microliters of Evans blue dye were used at the concentration of 0.20 mg/ml (200ug/1000ul). This is an excessive amount for tail vein injections, considering mouse weights around 30-40g. This could be a potential cause of mortality in animals. This concern needs to be resolved to identify the best possible dosage volume and concentration for the future application of this technique in mice. Also, references are needed to justify the Evans blue dosage volume and concentration.

E-Are the videos of the clasping test available for each animal? If not already done so, they can be evaluated blindly by two experimenters to confirm if their scores match the scale values reported in the study.

4-Discussion, add to the discussion section,

A-Authors mentioned that this technique has not been used in mice previously. There are studies that use the photo thrombotic technique in mice (i.e. Labat-gest V, Tomasi S. Photothrombotic Ischemia: A Minimally Invasive and Reproducible Photochemical Cortical Lesion Model for Mouse Stroke Studies). This technique presented is less invasive and reaching the MCA distal branch via a small bone drill seems minimally invasive compared to previously described techniques. I think describing the differences between the commonly used photothrombotic techniques and this current method is important and needs to be discussed.

B-Presented data is convincing indicating this model injures the MCA territory. The images that are shown seem to result in severe injury. Please comment on the severity of the injury and explain the degree of damage in this model. Is it moderate or severe? Is it possible to control the severity of injury?

C-Given the severity of the stroke-induced, do the authors think that any treatment strategy (neuroprotective strategies) would be possible?

D-One other limitation of this model is that this is an irreversible complete obstruction of the MCA resulting in stroke. Thus, studying the immediate reperfusion injury seems to be a limitation. Please clarify and revise accordingly. The authors mention that the recanalization via catheterization to study the reperfusion injury can be performed, please comment on how feasible this intervention is in this model.

E-Also please comment on the application of this model to other experimental animals, such as larger animal models and younger age in rodents such as neonatal rodents.

## References

[B1] Adam I, Cecchini G, Fanelli D, Kreuz T, Livi R, di Volo M, Allegra Mascaro AL, Conti E, Scaglione A, Silvestri L, Pavone FS (2020) Inferring network structure and local dynamics from neuronal patterns with quenched disorder. Chaos Solitons Fractals 140:110235. 10.1016/j.chaos.2020.110235

[B2] Alia C, Cangi D, Massa V, Salluzzo M, Vignozzi L, Caleo M, Spalletti C (2021) Cell-to-cell interactions mediating functional recovery after stroke. Cells 10:3050. 10.3390/cells10113050 34831273 PMC8623942

[B3] Allegra Mascaro AL, Letizia A, Conti E, Lai S, Di Giovanna AP, Spalletti C, Alia C, Panarese A, Scaglione A, Sacconi L, Micera S, Caleo M, Pavone FS (2019) Combined rehabilitation promotes the recovery of structural and functional features of healthy neuronal networks after stroke. Cell Rep 28:3474–3485.e6. 10.1016/j.celrep.2019.08.062 31553915

[B4] Balbi M, Vanni MP, Silasi G, Sekino Y, Bolanos L, LeDue JM, Murphy TH (2017) Targeted ischemic stroke induction and mesoscopic imaging assessment of blood flow and ischemic depolarization in awake mice. Neurophotonics 4:e035001. 10.1117/1.NPh.4.3.035001 28721356 PMC5512458

[B5] Balkaya M, Kröber JM, Rex A, Endres M (2013) Assessing post-stroke behavior in mouse models of focal ischemia. J Cereb Blood Flow Metab 33:330–338. 10.1038/jcbfm.2012.185 23232947 PMC3587814

[B6] Belayev L, Busto R, Zhao W, Ginsberg MD (1996) Quantitative evaluation of blood-brain barrier permeability following middle cerebral artery occlusion in rats. Brain Res 739:88–96. 10.1016/S0006-8993(96)00815-3 8955928

[B7] Braeuninger S, Kleinschnitz C (2009) Rodent models of focal cerebral ischemia: procedural pitfalls and translational problems. Exp Transl Stroke Med 1:8. 10.1186/2040-7378-1-8 20150986 PMC2820446

[B8] Candelario-Jalil E, Dijkhuizen RM, Magnus T (2022) Neuroinflammation, stroke, blood-brain barrier dysfunction, and imaging modalities. Stroke 53:1473–1486. 10.1161/STROKEAHA.122.036946 35387495 PMC9038693

[B9] Carmichael ST (2005) Rodent models of focal stroke: size, mechanism, and purpose. NeuroRx 2:396–409. 10.1602/neurorx.2.3.396 16389304 PMC1144484

[B10] Cecchini G, Scaglione A, Allegra Mascaro AL, Checcucci C, Conti E, Adam I, Fanelli D, Livi R, Saverio Pavone F, Kreuz T (2021) Cortical propagation tracks functional recovery after stroke. PLoS Comput Biol 17:e1008963. 10.1371/journal.pcbi.1008963 33999967 PMC8159272

[B11] Clark TA, Sullender C, Kazmi SM, Speetles BL, Williamson MR, Palmberg DM, Dunn AK, Jones TA (2019) Artery targeted photothrombosis widens the vascular penumbra, instigates peri-infarct neovascularization and models forelimb impairments. Sci Rep 9:2323. 10.1038/s41598-019-39092-7 30787398 PMC6382883

[B12] Conti E, Piccardi B, Sodero A, Tudisco L, Lombardo I, Fainardi E, Nencini P, Sarti C, Allegra Mascaro AL, Baldereschi M (2021) Translational stroke research review: using the mouse to model human futile recanalization and reperfusion injury in ischemic brain tissue. Cells 10:3308. 10.3390/cells10123308 34943816 PMC8699609

[B13] Conti E, Scaglione A, de Vito G, Calugi F, Pasquini M, Pizzorusso T, Micera S, Allegra Mascaro AL, Pavone FS (2022) Combining optogenetic stimulation and motor training improves functional recovery and perilesional cortical activity. Neurorehabil Neural Repair 36:107–118. 10.1177/15459683211056656 34761714

[B14] Dietrich WD, Watson BD, Busto R, Ginsberg MD, Bethea JR (1987) Photochemically induced cerebral infarction. I. Early microvascular alterations. Acta Neuropathol 72:315–325. 10.1007/BF00687262 3577687

[B15] Fernandez-Lopez D, Faustino J, Daneman R, Zhou L, Lee SY, Derugin N, Wendland MF, Vexler ZS (2012) Blood–brain barrier permeability is increased after acute adult stroke but not neonatal stroke in the rat. J Neurosci 32:9588–9600. 10.1523/JNEUROSCI.5977-11.2012 22787045 PMC3539825

[B16] Gonzalez CLR, Kolb B (2003) A comparison of different models of stroke on behaviour and brain morphology. Eur J Neurosci 18:1950–1962. 10.1046/j.1460-9568.2003.02928.x 14622227

[B17] Guyenet SJ, Furrer SA, Damian VM, Baughan TD, La Spada AR, Garden GA (2010) A simple composite phenotype scoring system for evaluating mouse models of cerebellar ataxia. J Vis Exp (39):e1787. 10.3791/1787 PMC3121238 20495529

[B18] Howells DW, Porritt MJ, Rewell SSJ, O’Collins V, Sena ES, van der Worp HB, Traystman RJ, Macleod MR (2010) Different strokes for different folks: the rich diversity of animal models of focal cerebral ischemia. J Cereb Blood Flow Metab 30:1412–1431. 10.1038/jcbfm.2010.66 20485296 PMC2949237

[B19] Ishrat T, Sayeed I, Atif F, Stein DG (2009) Effects of progesterone administration on infarct volume and functional deficits following permanent focal cerebral ischemia in rats. Brain Res 1257:94–101. 10.1016/j.brainres.2008.12.048 19135987 PMC2656912

[B20] Jia J-M, Peng C, Wang Y, Zheng J, Ge W-P (2018) Control of occlusion of middle cerebral artery in perinatal and neonatal mice with magnetic force. Mol Brain 11:47. 10.1186/s13041-018-0389-0 30157965 PMC6114863

[B21] Kanemitsu H, Nakagomi T, Tamura A, Tsuchiya T, Kono G, Sano K (2002) Differences in the extent of primary ischemic damage between middle cerebral artery coagulation and intraluminal occlusion models. J Cereb Blood Flow Metab 22:1196–1204. 10.1097/01.wcb.0000037992.07114.95 12368658

[B22] Kenne E, Erlandsson A, Lindbom L, Hillered L, Clausen F (2012) Neutrophil depletion reduces edema formation and tissue loss following traumatic brain injury in mice. J Neuroinflammation 9:17. 10.1186/1742-2094-9-17 22269349 PMC3292978

[B23] Knight RA, Han Y, Nagaraja TN, Whitton P, Ding J, Chopp M, Seyfried DM (2008) Temporal MRI assessment of intracerebral hemorrhage in rats. Stroke 39:2596–2602. 10.1161/STROKEAHA.107.506683 18635862 PMC3980853

[B24] Kreuz T, Senocrate F, Cecchini G, Checcucci C, Mascaro ALA, Conti E, Scaglione A, Pavone FS (2022) Latency correction in sparse neuronal spike trains. J Neurosci Methods 381:109703. 10.1016/j.jneumeth.2022.109703 36075286 PMC9554712

[B25] Labat-gest V, Tomasi S (2013) Photothrombotic ischemia: a minimally invasive and reproducible photochemical cortical lesion model for mouse stroke studies. J Vis Exp (76):50370. 10.3791/50370 23770844 PMC3727176

[B26] Li H, Zhang N, Lin H-Y, Yu Y, Cai Q-Y, Ma L, Ding S (2014) Histological, cellular and behavioral assessments of stroke outcomes after photothrombosis-induced ischemia in adult mice. BMC Neurosci 15:58. 10.1186/1471-2202-15-58 24886391 PMC4039545

[B27] Macrae IM (2011) Preclinical stroke research—advantages and disadvantages of the most common rodent models of focal ischaemia. Br J Pharmacol 164:1062–1078. 10.1111/j.1476-5381.2011.01398.x 21457227 PMC3229752

[B28] Mascaro ALA, et al. (2020) Experimental and computational study on motor control and recovery after stroke: toward a constructive loop between experimental and virtual embodied neuroscience. Front Syst Neurosci 14:31. 10.3389/fnsys.2020.00031 32733210 PMC7359878

[B29] Matsuno H, Uematsu T, Umemura K, Takiguchi Y, Asai Y, Muranaka Y, Nakashima M (1993) A simple and reproducible cerebral thrombosis model in rats induced by a photochemical reaction and the effect of a plasminogen-plasminogen activator chimera in this model. J Pharmacol Toxicol Methods 29:165–173. 10.1016/1056-8719(93)90068-P 8364230

[B30] Matsushita H, Hijioka M, Hisatsune A, Isohama Y, Iwamoto S, Terasawa H, Katsuki H (2013) MRI-based analysis of intracerebral hemorrhage in mice reveals relationship between hematoma expansion and the severity of symptoms. PLoS One 8:e67691. 10.1371/journal.pone.0067691 23844065 PMC3699642

[B31] Maxwell KA, Dyck RH (2005) Induction of reproducible focal ischemic lesions in neonatal mice by photothrombosis. Dev Neurosci 27:121–126. 10.1159/000085983 16046845

[B32] Menzies SA, Hoff JT, Betz AL (1992) Middle cerebral artery occlusion in rats: a neurological and pathological evaluation of a reproducible model. Neurosurgery 31:100–106. 10.1227/00006123-199207000-00014 1641086

[B33] Miedel CJ, Patton JM, Miedel AN, Miedel ES, Levenson JM (2017) Assessment of spontaneous alternation, novel object recognition and limb clasping in transgenic mouse models of amyloid-β and tau neuropathology. J Vis Exp (123):55523. 10.3791/55523 28605382 PMC5608159

[B34] Mies G, Ishimaru S, Xie Y, Seo K, Hossmann KA (1991) Ischemic thresholds of cerebral protein synthesis and energy state following middle cerebral artery occlusion in rat. J Cereb Blood Flow Metab 11:753–761. 10.1038/jcbfm.1991.132 1874807

[B35] Mora-Lee S, Salomé Sirerol-Piquer M, Gutiérrez-Pérez M, López T, Casado-Nieto M, Jauquicoam C, Abizanda G, Romaguera-Ros M, Gomez-Pinedo U, Prósper F, García-Verdugo J-M (2011) Histological and ultrastructural comparison of cauterization and thrombosis stroke models in immune-deficient mice. J Inflamm (Lond) 8:28. 10.1186/1476-9255-8-28 22008614 PMC3221623

[B36] Reglodi D, Somogyvari-Vigh A, Maderdrut JL, Vigh S, Arimura A (2000) Postischemic spontaneous hyperthermia and its effects in middle cerebral artery occlusion in the rat. Exp Neurol 163:399–407. 10.1006/exnr.2000.7367 10833314

[B37] Rosenberg GA, Estrada EY, Dencoff JE (1998) Matrix metalloproteinases and TIMPs are associated with blood-brain barrier opening after reperfusion in rat brain. Stroke 29:2189–2195. 10.1161/01.STR.29.10.2189 9756602

[B38] Saniabadi AR, Umemura K, Matsumoto N, Sakuma S, Nakashima M (1995) Vessel wall injury and arterial thrombosis induced by a photochemical reaction. Thromb Haemost 73:868–872. 10.1055/s-0038-1653883 7482418

[B39] Saunders NR, Dziegielewska KM, Møllgård K, and MD Habgood (2015) Markers for blood-brain barrier integrity: how appropriate is Evans blue in the twenty-first century and what are the alternatives? Front Neurosci 9:385. 10.3389/fnins.2015.00385 26578854 PMC4624851

[B40] Scaglione A, Conti E, Allegra Mascaro AL, Pavone FS (2022) Tracking the effect of therapy with single-trial based classification after stroke. Front Syst Neurosci 16:840922. 10.3389/fnsys.2022.840922 35602972 PMC9114305

[B41] Shen J, Ishii Y, Xu G, Dang TC, Hamashima T, Matsushima T, Yamamoto S, Hattori Y, Takatsuru Y, Nabekura J, Sasahara M (2012) PDGFR-β as a positive regulator of tissue repair in a mouse model of focal cerebral ischemia. J Cereb Blood Flow Metab 32:353–367. 10.1038/jcbfm.2011.136 21952111 PMC3272602

[B42] Sheth KN, Kimberly WT, Elm JJ, Kent TA, Mandava P, Yoo AJ, Thomalla G, Campbell B, Donnan GA, Davis SM, Albers GW, Jacobson S, Simard JM, Stern BJ (2014) Pilot study of intravenous glyburide in patients with a large ischemic stroke. Stroke 45:281–283. 10.1161/STROKEAHA.113.003352 24193798 PMC4235339

[B43] Sheth KN, Elm JJ, Molyneaux BJ, Hinson H, Beslow LA, Sze GK, Ostwaldt A-C, del Zoppo GJ, Simard JM, Jacobson S, Kimberly WT (2016) Safety and efficacy of intravenous glyburide on brain swelling after large hemispheric infarction (GAMES-RP): a randomised, double-blind, placebo-controlled phase 2 trial. Lancet Neurol 15:1160–1169. 10.1016/S1474-4422(16)30196-X 27567243

[B44] Sheth KN, Petersen NH, Cheung K, Elm JJ, Hinson HE, Molyneaux BJ, Beslow LA, Sze GK, Simard JM, Kimberly WT (2018) Long-term outcomes in patients aged ≤70 years with intravenous glyburide from the phase II GAMES-RP Study of Large Hemispheric Infarction. Stroke 49:1457–1463. 10.1161/STROKEAHA.117.020365 29789393 PMC6192530

[B45] Shih AY, Nishimura N, Nguyen J, Friedman B, Lyden PD, Schaffer CB, Kleinfeld D (2013) Optically induced occlusion of single blood vessels in rodent neocortex. Cold Spring Harb Protoc 2013:1153–1160. 10.1101/pdb.prot079509 24298038

[B46] Simard JM, Sheth KN, Kimberly WT, Stern BJ, del Zoppo GJ, Jacobson S, Gerzanich V (2014) Glibenclamide in cerebral ischemia and stroke. Neurocrit Care 20:319–333. 10.1007/s12028-013-9923-1 24132564 PMC3954940

[B47] Stoll G, Kleinschnitz C, Meuth SG, Braeuninger S, Ip CW, Wessig C, Nölte I, Bendszus M (2009) Transient widespread blood-brain barrier alterations after cerebral photothrombosis as revealed by gadofluorine M-enhanced magnetic resonance imaging. J Cereb Blood Flow Metab 29:331–341. 10.1038/jcbfm.2008.129 18957988

[B48] Sugimori H, Yao H, Ooboshi H, Ibayashi S, Iida M (2004) Krypton laser-induced photothrombotic distal middle cerebral artery occlusion without craniectomy in mice. Brain Res Brain Res Protoc 13:189–196. 10.1016/j.brainresprot.2004.06.001 15296857

[B49] Sunil S, Erdener SE, Lee BS, Postnov D, Tang J, Kura S, Cheng X, Chen IA, Boas DA, Kılıç K (2020) Awake chronic mouse model of targeted pial vessel occlusion via photothrombosis. Neurophotonics 7:e015005. 10.1117/1.NPh.7.1.015005 32042854 PMC6992450

[B50] Taheri S, Candelario-Jalil E, Estrada EY, Rosenberg GA (2009) Spatiotemporal correlations between blood-brain barrier permeability and apparent diffusion coefficient in a rat model of ischemic stroke. PLoS One 4:e6597. 10.1371/journal.pone.0006597 19668371 PMC2719093

[B51] Takamatsu H, Tatsumi M, Nitta S, Ichise R, Muramatsu K, Iida M, Nishimura S, Umemura K (2002) Time courses of progress to the chronic stage of middle cerebral artery occlusion models in rats. Exp Brain Res 146:95–102. 10.1007/s00221-002-1147-0 12192583

[B52] Wafa HA, Wolfe CDA, Emmett E, Roth GA, Johnson CO, Wang Y (2020) Burden of stroke in Europe: thirty-year projections of incidence, prevalence, deaths, and disability-adjusted life years. Stroke 51:2418–2427. 10.1161/STROKEAHA.120.029606 32646325 PMC7382540

[B53] Watson BD, Dietrich WD, Prado R, Ginsberg MD (1987) Argon laser-induced arterial photothrombosis. Characterization and possible application to therapy of arteriovenous malformations. J Neurosurg 66:748–754. 10.3171/jns.1987.66.5.0748 3572500

[B54] Watson BD, Prado R, Veloso A, Brunschwig J-P, Dietrich WD (2002) Cerebral blood flow restoration and reperfusion injury after ultraviolet laser–facilitated middle cerebral artery recanalization in rat thrombotic stroke. Stroke 33:428–434. 10.1161/hs0202.102730 11823647

[B55] Yang J, Li Q, Wang Z, Qi C, Han X, Lan X, Wan J, Wang W, Zhao X, Hou Z, Gao C, Carhuapoma JR, Mori S, Zhang J, Wang J (2017) Multimodality MRI assessment of grey and white matter injury and blood-brain barrier disruption after intracerebral haemorrhage in mice. Sci Rep 7:40358. 10.1038/srep40358 28084426 PMC5234017

[B56] Yao H, Sugimori H, Fukuda K, Takada J, Ooboshi H, Kitazono T, Ibayashi S, Iida M (2003) Photothrombotic middle cerebral artery occlusion and reperfusion laser system in spontaneously hypertensive rats. Stroke 34:2716–2721. 10.1161/01.STR.0000094730.38343.73 14576380

[B57] Zhao B-Q, Suzuki Y, Kondo K, Kawano K-I, Ikeda Y, Umemura K (2002) A novel MCA occlusion model of photothrombotic ischemia with cyclic flow reductions: development of cerebral hemorrhage induced by heparin. Brain Res Brain Res Protoc 9:85–92. 10.1016/s1385-299x(01)00124-6 12034327

[B58] Zhao Q, Memezawa H, Smith M-L, Siejö BK (1994) Hyperthermia complicates middle cerebral artery occlusion induced by an intraluminal filament. Brain Res 649:253–259. 10.1016/0006-8993(94)91071-5 7953639

