# Supplementary figures and images for: Photothrombotic Middle Cerebral Artery Occlusion in Mice: A Novel Model of Ischemic Stroke
Source: eNeuro. 2023 Feb 7;10(2):ENEURO.0244-22.2022. doi: 10.1523/ENEURO.0244-22.2022 (PMC9910575; doi:10.1523/ENEURO.0244-22.2022)

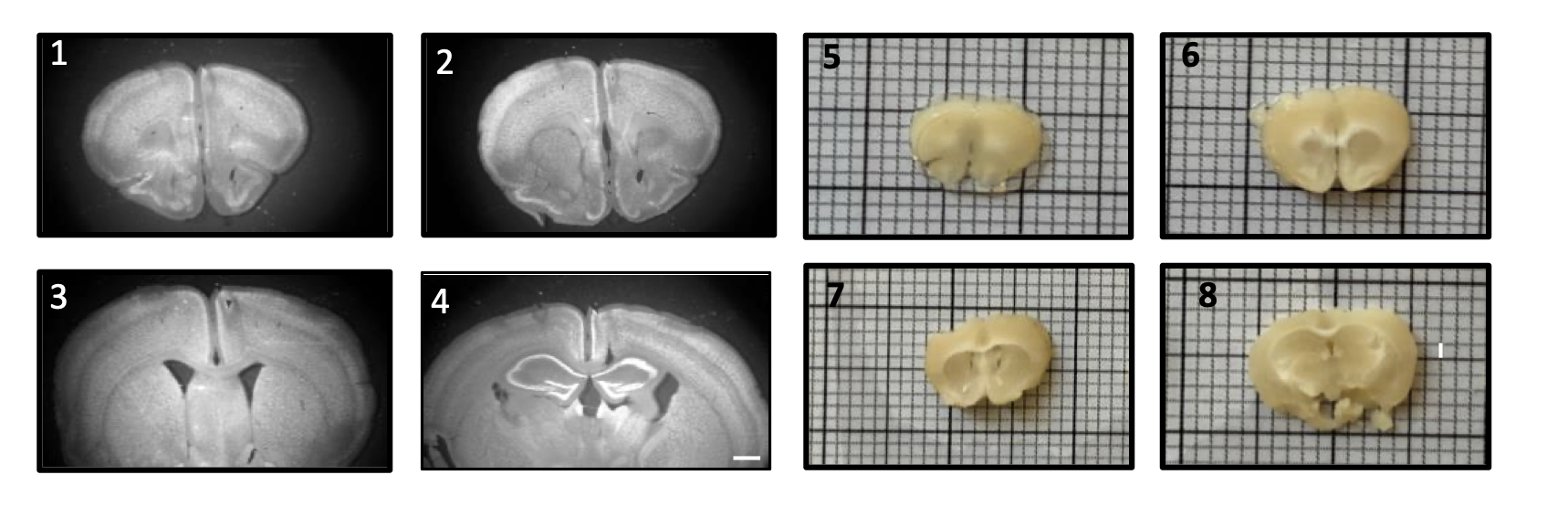

Supplement: Figure 1-1 — Sham mice ex vivo does not show sign of tissue suffering. On the right, panels 1–4 show representative coronal brain slices (100 μm thick) labeled with NeuN antibody 1 week after surgery. The ex vivo analysis does not find regions of tissue suffering or necrosis due to craniotomy or laser irradiation. Scale bar, 1 mm. On the left, panels 5–8 show representative coronal brain slices (1 mm thick), 24 h after surgery and intravenous injection of Evans Blue dye. The absence of blue staining highlights that the surgery followed by green laser illumination does not induce BBB permeability alterations. Download Figure 1-1, TIF file. [file enu-eN-MNT-0244-22-s01.tif]

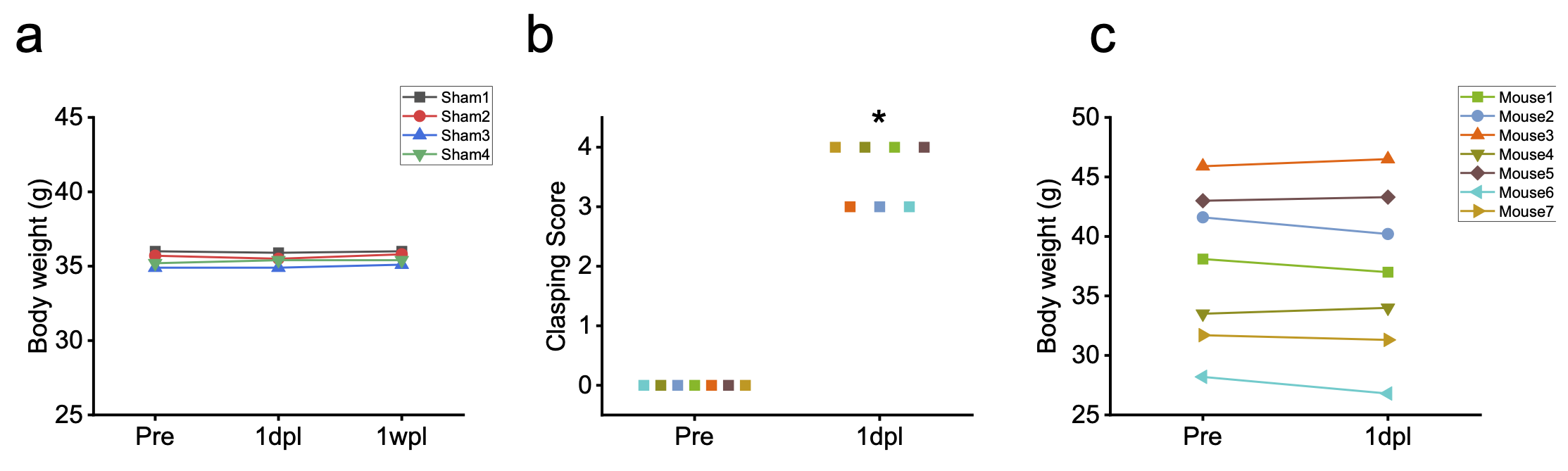

Supplement: Figure 2-1 — a, Body weight evaluation of Sham group at three different time points Pre, 1dpl, and 1wpl, respectively. b, As observed in MCAPT mice, the clasping reflex revealed a tendency to higher clasping behavior after stroke in the acute phase (1dpl) in the EB group as well as in the MCAPT group. *p value based on one-way repeated-measures ANOVA followed by post hoc Tukey’s correction: Pre-1dpl, p = 0.00002. c, Body weight monitoring does not highlight any alteration after the MCA occlusion. Download Figure 2-1, TIF file. [file enu-eN-MNT-0244-22-s02.tif]

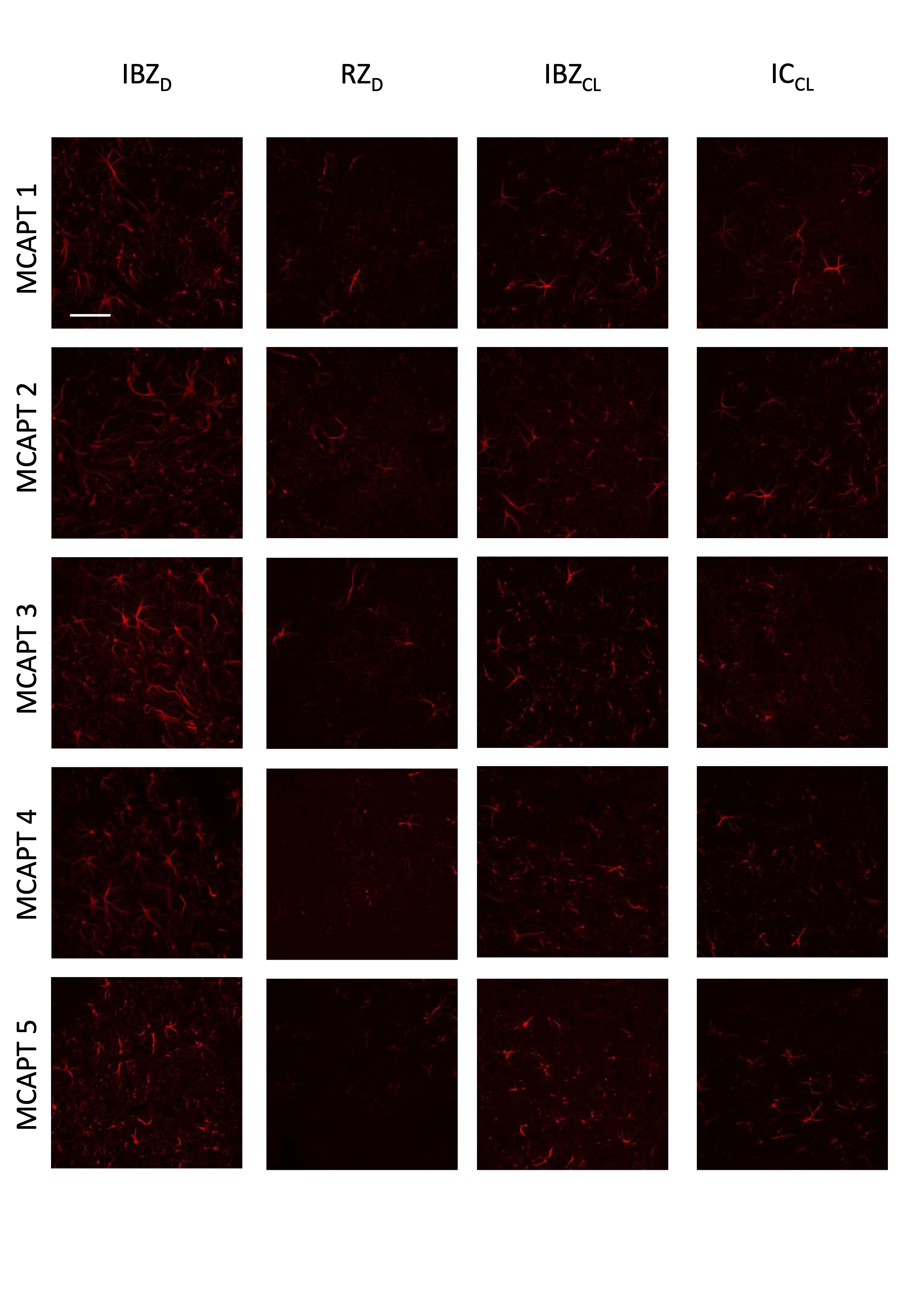

Supplement: Figure 4-1 — GFAP analysis. Representative images of GFAP-labeled astrocytes in the four different regions of interest (IBZIL, RZIL, IBZCL, ICCL) for each mouse. Download Figure 4-1, TIF file. [file enu-eN-MNT-0244-22-s13.tif]

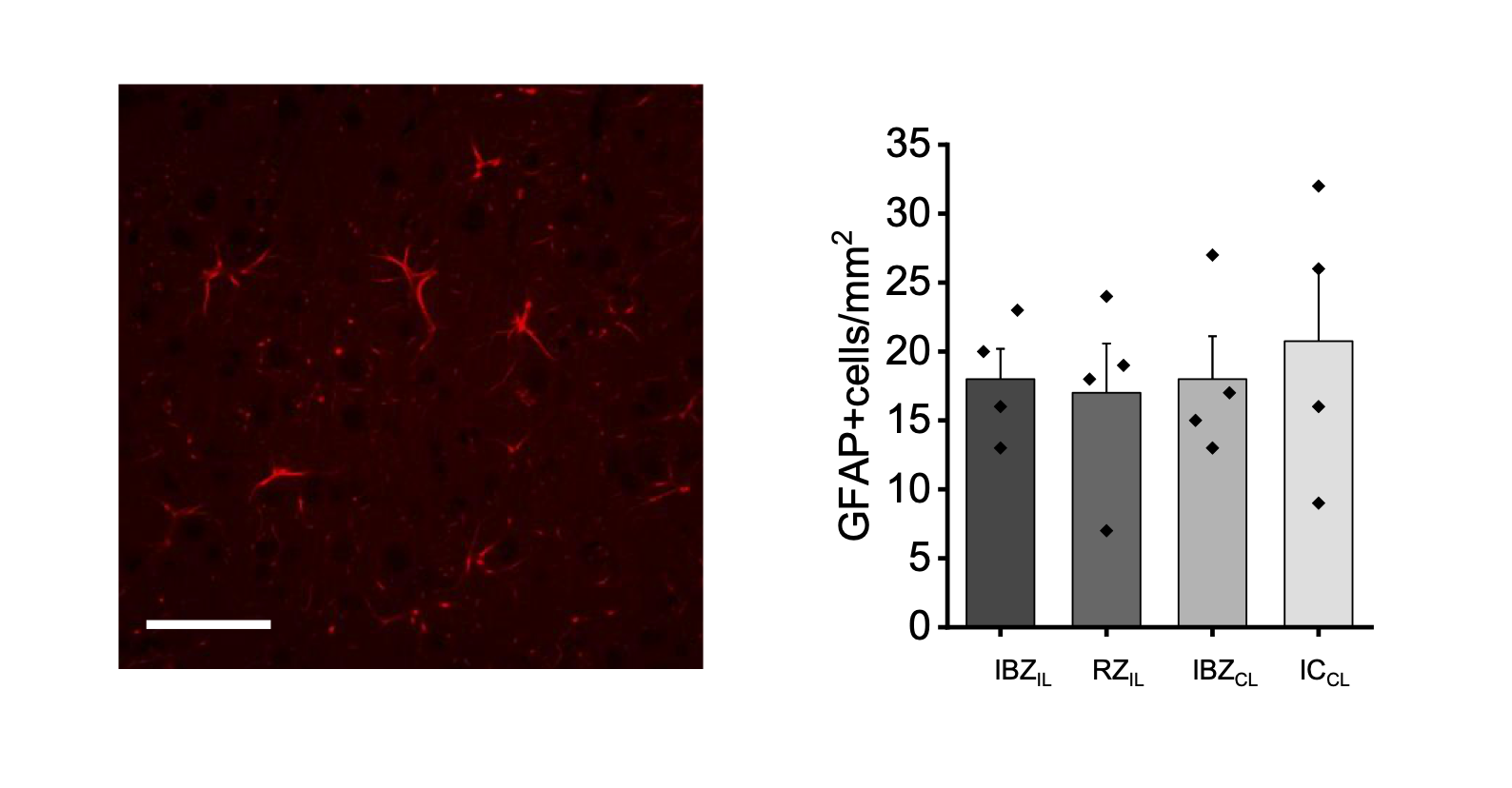

Supplement: Figure 4-2 — Astrocytes density in Sham mice: on the left, a representative image of anti-GFAP-labeled astrocytes. Scale bar, 45 μm. The graph on the right shows the density (average ± SEM) of GFAP-positive cells in the 4 ROIs (IBZIL = 18 ± 2.2; RZIL = 17 ± 3.6; IBZCL = 18 ± 3.1; ICCL = 20.7 ± 5.1). Download Figure 4-2, TIF file. [file enu-eN-MNT-0244-22-s03.tif]

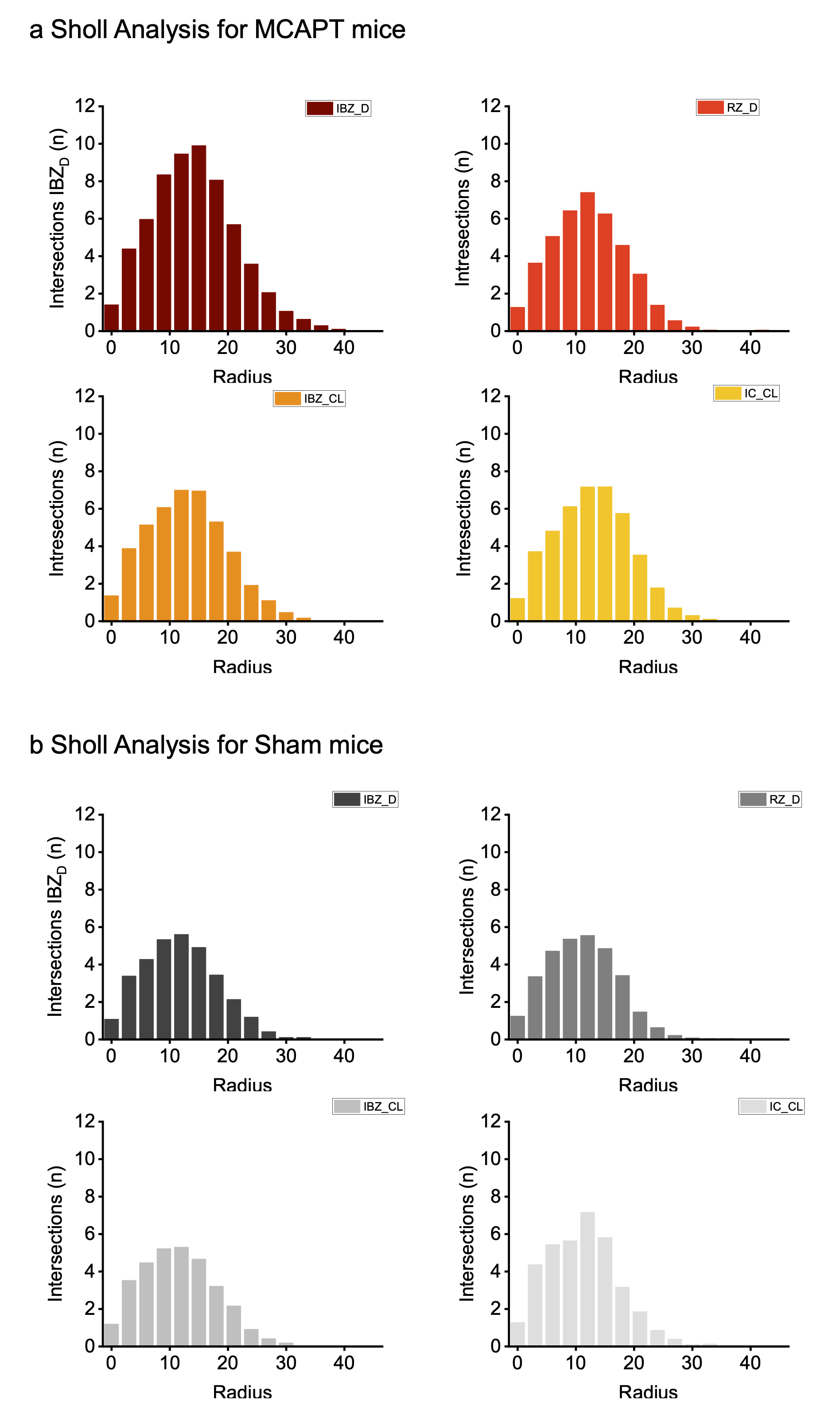

Supplement: Figure 5-1 — Sholl analysis in MCAPT and Sham mice. The graphs show the distribution of the number of intersections for each radius in the 4 ROIs, color coded as in Figure 5. Download Figure 5-1, TIF file. [file enu-eN-MNT-0244-22-s04.tif]

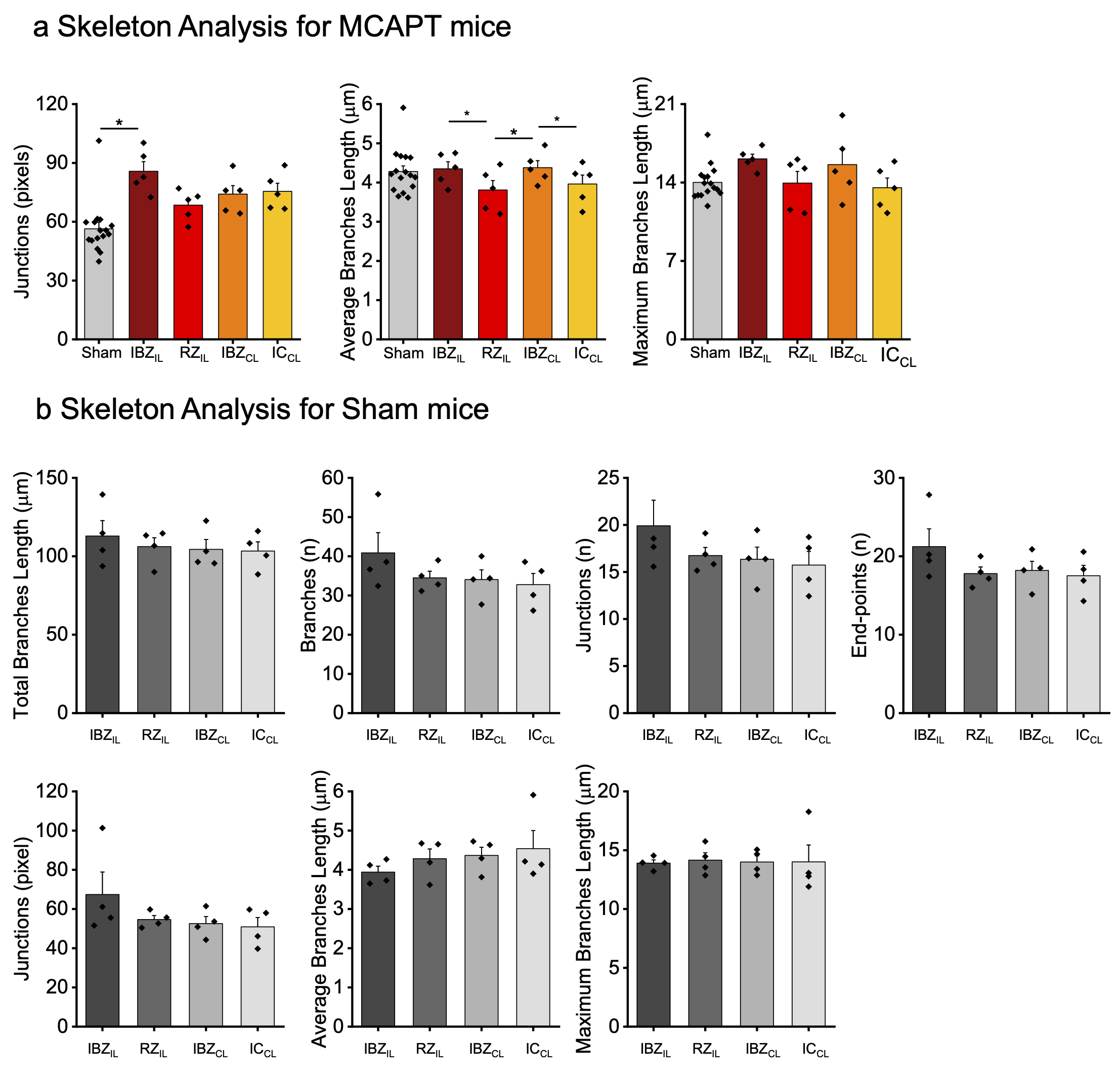

Supplement: Figure 5-2 — a, b, Skeleton analysis of astrocytes in MCAPT mice (a) and Sham (b) mice. All the parameters evaluated in the 4 ROIs are shown as the average ± SEM. The intergroup statistical analysis was performed through a two-way repeated-measures ANOVA followed by a post hoc Tukey’s HSD test (see Extended Data Table 5-4). The intragroup statistical analysis was performed through a one-way repeated-measures ANOVA followed by a post hoc Tukey’s HSD test (see Extended Data Tables 5-5, Tables 5-6). a, Junctions (pixel): Sham = 56.37 ± 3.37; IBZIL = 85.74 ± 4.93; RZIL = 68.45 ± 3.5; IBZCL = 74.01 ± 4.36 ICCL = 75.47 ± 4.2; intergroup analysis: IBZIL MCAPT-Sham, p = 0.00009. Average length of branches (μm): Sham = 4.28 ± 0.14; IBZIL = 4.35 ± 0.18; RZIL = 3.81 ± 0.24; IBZCL = 4.38 ± 0.18; ICCL = 3.96 ± 0.23; intergroup analysis: IBZIL MCAPT-Sham, p = 0.03; intragroup analysis: IBZIL-RZIL, p = 0.01; IBZCL-RZIL, p = 0.007; IBZCL-ICCL, p = 0.05. Maximum length of branches (μm): Sham = 14.01 ± 0.37; IBZIL = 16.11 ± 0.42; RZIL = 13.95 ± 1.04; IBZCL = 15.6 ± 1.37; ICCL = 13.53 ± 0.87. b, Total length of branches (μm): Sham, IBZIL = 112.914 ± 9.819; RZIL = 106.157 ± 5.648; IBZCL = 104.43 ± 6.303; ICCL = 193.352 ± 5.869. Number of astrocytes in branches (average ± SEM) in the 4 ROIs: IBZIL = 40.87 ± 5.15; RZIL = 34.49 ± 1.7 IBZCL = 34.05 ± 2.51; ICCL = 32.76 ± 2.84. Number of astrocytes in junctions: IBZIL = 19.91 ± 2.71; RZIL = 16.74 ± 0.87; IBZCL = 16.35 ± 1.29; ICCL = 15.73 ± 1.45. Number of astrocytes at end points in the 4 ROIs: IBZIL = 21.23 ± 2.28; RZIL = 17.79 ± 0.84; IBZCL = 18.19 ± 1.18; ICCL = 17.519 ± 1.317. Junctions (pixels): IBZIL = 67.39 ± 11.48; RZIL = 54.63 ± 2.02; IBZCL = 52.56 ± 3.55; ICCL = 50.88 ± 4.79. Average length of branches (μm): IBZIL = 3.9 ± 0.15; RZIL = 4.3 ± 0.25; IBZCL = 4.37 ± 0.21; ICCL = 4.54 ± 0.47. Maximum length of branches (μm): IBZIL = 13.90 ± 0.27; RZIL = 14.15 ± 0.62; IBZCL = 14 ± 0.51; ICCL = 14 ± 1.44. Download Figure 5-2, TIF file. [file enu-eN-MNT-0244-22-s05.tif]
