# Supplementary material for: Photothrombotic Middle Cerebral Artery Occlusion in Mice: A Novel Model of Ischemic Stroke
Source: eNeuro. 2023 Feb 7;10(2):ENEURO.0244-22.2022. doi: 10.1523/ENEURO.0244-22.2022 (PMC9910575; doi:10.1523/ENEURO.0244-22.2022)
Supplement: Table 4-1 — Astrocytes density intragroup (Sham and MCAPT) and intergroups comparison. One-way repeated-measures ANOVA followed by Tukey’s test was used for intragroup comparison. Two-way repeated-measures ANOVA followed by Tukey’s test was used for intergroup comparison. Colored cells indicate p-values < 0.05. Download Table 4-1, DOC file. [file enu-eN-MNT-0244-22-s06.doc]

| **Density** | **Sham vs Stroke** |
| --- | --- |
| **IBZ_IL_** | 6.88E-07 |
| **RZ_IL_** | 0.09458 |
| **IBZ_CL_** | 0.0043 |
| **IC_CL_** | 0.05908 |

| **Density** | **Sham** | **MCAPT** |
| --- | --- | --- |
| **IBZ_IL_-RZ_IL_** | 0.9973 | 0.00721 |
| **IBZ_IL_-IBZ_CL_** | 1 | 0.02508 |
| **IBZ_IL_-IC_CL_** | 0.9973 | 0.01278 |
| **RZ_IL_-IBZ_CL_** | 0.94974 | 0.88117 |
| **RZ_IL_-IC_CL_** | 0.88539 | 0.99387 |
| **IBZ_CL_-IC_CL_** | 0.94974 | 0.96503 |
