# Supplementary material for: Photothrombotic Middle Cerebral Artery Occlusion in Mice: A Novel Model of Ischemic Stroke
Source: eNeuro. 2023 Feb 7;10(2):ENEURO.0244-22.2022. doi: 10.1523/ENEURO.0244-22.2022 (PMC9910575; doi:10.1523/ENEURO.0244-22.2022)
Supplement: Table 5-1 — Intragroup (MCAPT) comparison of Sholl analysis in different regions of the cortex. Two-way repeated-measures ANOVA followed by Tukey’s test. Colored cells indicate p-values < 0.05. Download Table 5-1, DOC file. [file enu-eN-MNT-0244-22-s07.doc]

| **Sholl** | **Bin1** | **Bin2** | **Bin3** | **Bin4** | **Bin5** | **Bin6** | **Bin7** | **Bin8** |
| --- | --- | --- | --- | --- | --- | --- | --- | --- |
| **IBZ_IL_-RZ_IL_** | 0.99835 | 0.91959 | 0.95037 | 0.6026 | 0.66948 | 3.42E-05 | 2.51E-08 | 8.65E-07 |
| **IBZ_IL_-IBZ_CL_** | 0.99308 | 0.99974 | 0.99581 | 0.38816 | 0.66232 | 0.0605 | 0.00638 | 0.01349 |
| **IBZ_IL_-IC_CL_** | 0.99082 | 0.97221 | 0.67217 | 0.19198 | 0.32152 | 0.07273 | 0.00598 | 1.72E-04 |
| **RZ_IL_-IBZ_CL_** | 0.9996 | 0.94684 | 0.87089 | 0.98545 | 1 | 0.14003 | 0.02244 | 0.07763 |
| **RZ_IL_-IC_CL_** | 0.99919 | 0.99707 | 0.93386 | 0.87224 | 0.93896 | 0.11916 | 0.02378 | 0.63434 |
| **IBZ_CL_-IC_CL_** | 0.99999 | 0.98567 | 0.52805 | 0.97638 | 0.94226 | 0.99985 | 1 | 0.6106 |
| **Sholl** | **Bin9** | **Bin10** | **Bin11** | **Bin12** | **Bin13** | **Bin14** | **Bin15** | **Bin16** |
| **IBZ_IL_-RZ_IL_** | 6.82E-06 | 0.00422 | 0.18501 | 0.65502 | 0.94518 | 0.99969 | 0.99979 | 0.99999 |
| **IBZ_IL_-IBZ_CL_** | 0.02925 | 0.40918 | 0.64282 | 0.85752 | 0.96155 | 0.99958 | 0.99993 | 0.99998 |
| **IBZ_IL_-IC_CL_** | 4.10E-04 | 0.01352 | 0.27485 | 0.73549 | 0.9411 | 0.99838 | 0.99993 | 0.99998 |
| **RZ_IL_-IBZ_CL_** | 0.11592 | 0.24365 | 0.83404 | 0.98354 | 0.9999 | 1 | 0.99902 | 0.99998 |
| **RZ_IL_-IC_CL_** | 0.76774 | 0.98339 | 0.9965 | 0.99917 | 1 | 0.99988 | 0.99902 | 0.99998 |
| **IBZ_CL_-IC_CL_** | 0.58059 | 0.43493 | 0.92294 | 0.99564 | 0.99981 | 0.99992 | 1 | 1 |
