# Supplementary material for: Photothrombotic Middle Cerebral Artery Occlusion in Mice: A Novel Model of Ischemic Stroke
Source: eNeuro. 2023 Feb 7;10(2):ENEURO.0244-22.2022. doi: 10.1523/ENEURO.0244-22.2022 (PMC9910575; doi:10.1523/ENEURO.0244-22.2022)
Supplement: Table 5-2 — Intragroup (Sham) comparison of Sholl analysis in different regions of the cortex. Two-way repeated-measures ANOVA followed by Tukey’s test. Colored cells indicate p-values < 0.05. Download Table 5-2, DOC file. [file enu-eN-MNT-0244-22-s08.doc]

| **Sholl** | **Bin1** | **Bin2** | **Bin3** | **Bin4** | **Bin5** | **Bin6** | **Bin7** | **Bin8** |
| --- | --- | --- | --- | --- | --- | --- | --- | --- |
| **IBZ_IL_-RZ_IL_** | 0.99323 | 0.99997 | 0.891 | 0.99997 | 0.99974 | 0.99974 | 0.99997 | 0.70686 |
| **IBZ_IL_-IBZ_CL_** | 0.99796 | 0.99605 | 0.98935 | 0.99796 | 0.96086 | 0.97791 | 0.98428 | 0.99997 |
| **IBZ_IL_-IC_CL_** | 0.98844 | 0.39237 | 0.24289 | 0.95882 | 0.06546 | 0.47197 | 0.9718 | 0.97013 |
| **RZ_IL_-IBZ_CL_** | 0.99974 | 0.99323 | 0.97791 | 0.99605 | 0.97791 | 0.98935 | 0.98935 | 0.67961 |
| **RZ_IL_-IC_CL_** | 0.99994 | 0.36722 | 0.65187 | 0.96839 | 0.0524 | 0.41827 | 0.97929 | 0.92369 |
| **IBZ_CL_-IC_CL_** | 0.99895 | 0.52757 | 0.40264 | 0.90482 | 0.01752 | 0.25475 | 0.99981 | 0.96086 |
| **Sholl** | **Bin9** | **Bin10** | **Bin11** | **Bin12** | **Bin13** | **Bin14** | **Bin15** | **Bin16** |
| **IBZ_IL_-RZ_IL_** | 0.80811 | 0.98935 | 0.99997 | 0.99997 | 0.99974 | 0.99997 | 0.99997 | 1 |
| **IBZ_IL_-IBZ_CL_** | 0.97013 | 1 | 0.99605 | 1 | 1 | 1 | 1 | 1 |
| **IBZ_IL_-IC_CL_** | 0.95234 | 0.99997 | 0.99981 | 0.99825 | 1 | 1 | 1 | 1 |
| **RZ_IL_-IBZ_CL_** | 0.97013 | 0.98935 | 0.99796 | 0.99997 | 0.99974 | 0.99997 | 0.99997 | 1 |
| **RZ_IL_-IC_CL_** | 0.98312 | 0.99323 | 0.99998 | 0.9993 | 0.99974 | 0.99997 | 0.99997 | 1 |
| **IBZ_CL_-IC_CL_** | 0.99981 | 0.99997 | 0.99895 | 0.99825 | 1 | 1 | 1 | 1 |
