# Supplementary material for: Photothrombotic Middle Cerebral Artery Occlusion in Mice: A Novel Model of Ischemic Stroke
Source: eNeuro. 2023 Feb 7;10(2):ENEURO.0244-22.2022. doi: 10.1523/ENEURO.0244-22.2022 (PMC9910575; doi:10.1523/ENEURO.0244-22.2022)
Supplement: Table 5-3 — Intergroup (MCAPT and Sham) comparison of Sholl analysis for each region of the cortex. Two-way repeated-measures ANOVA followed by Tukey’s test. Colored cells indicate p-values < 0.05. Download Table 5-3, DOC file. [file enu-eN-MNT-0244-22-s09.doc]

| **Sholl** | **Bin1** | **Bin2** | **Bin3** | **Bin4** | **Bin5** | **Bin6** | **Bin7** | **Bin8** |
| --- | --- | --- | --- | --- | --- | --- | --- | --- |
| **IBZ_IL_ Sham - Stroke** | 0.77594 | 0.06529 | 0.83279 | 0.19996 | 0.16493 | 0.04678 | 0.02303 | 0.10778 |
| **RZ_IL_ Sham - Stroke** | 0.00686 | 0.00686 | 1.89E-04 | 1.89E-04 | 0.01708 | 0.36024 | 0.00599 | 0.3019 |
| **IBZ_CL_ Sham - Stroke** | 0.02764 | 0.05682 | 1.82E-05 | 2.22E-04 | 0.00259 | 9.17E-04 | 0.00228 | 0.05046 |
| **IC_CL_ Sham - Stroke** | 0.00319 | 0.00104 | 0.05637 | 2.13E-04 | 0.00273 | 6.54E-04 | 0.00268 | 0.04494 |
| **Sholl** | **Bin9** | **Bin10** | **Bin11** | **Bin12** | **Bin13** | **Bin14** | **Bin15** | **Bin16** |
| **IBZ_IL_ Sham - Stroke** | 0.10778 | 0.06967 | 0.01843 | 0.01299 | 0.20205 | 0.84021 | 0.82001 | 1 |
| **RZ_IL_ Sham - Stroke** | 2.03E-04 | 0.03544 | 0.86161 | 0.97156 | 1 | 1 | 1 | 1 |
| **IBZ_CL_ Sham - Stroke** | 0.00168 | 0.02475 | 0.37311 | 0.98925 | 0.98785 | 0.98339 | 1 | 1 |
| **IC_CL_ Sham - Stroke** | 2.29E-04 | 0.03308 | 0.99994 | 1 | 1 |  | 1 | 1 |
