# Supplementary material for: Photothrombotic Middle Cerebral Artery Occlusion in Mice: A Novel Model of Ischemic Stroke
Source: eNeuro. 2023 Feb 7;10(2):ENEURO.0244-22.2022. doi: 10.1523/ENEURO.0244-22.2022 (PMC9910575; doi:10.1523/ENEURO.0244-22.2022)
Supplement: Table 5-4 — Intergroup (MCAPT and Sham) comparison of Skeleton analysis for each region of the cortex. Two-way repeated-measures ANOVA followed by Tukey’s test. Colored cells indicate p-values < 0.05. Download Table 5-4, DOC file. [file enu-eN-MNT-0244-22-s10.doc]

| **Skeleton** | **Total Branches Length** | **Branches** | **Junctions** | **End-points** |
| --- | --- | --- | --- | --- |
| **IBZ_IL_ Sham - Stroke** | 0.00203 | 1.95E-02 | 0.02715 | 0.01619 |
| **RZ_IL_ Sham - Stroke** | 0.70314 | 3.01E-01 | 0.39419 | 0.11748 |
| **IBZ_CL_ Sham - Stroke** | 0.02926 | 0.02656 | 0.0357 | 0.01517 |
| **IC_CL_ Sham - Stroke** | 0.09892 | 0.01194 | 0.01692 | 0.00515 |
| **Skeleton** | **Junction Pixel** | **Average Branches Length** | **Maximum Branches Length** |  |
| **IBZ_IL_ Sham - Stroke** | 9.56E-05 | 0.8786 | 0.5821 |  |
| **RZ_IL_ Sham - Stroke** | 0.15923 | 0.77953 | 1 |  |
| **IBZ_CL_ Sham - Stroke** | 0.00639 | 1 | 0.86556 |  |
| **IC_CL_ Sham - Stroke** | 0.00159 | 0.57994 | 0.9999 |  |
