# Supplementary material for: Photothrombotic Middle Cerebral Artery Occlusion in Mice: A Novel Model of Ischemic Stroke
Source: eNeuro. 2023 Feb 7;10(2):ENEURO.0244-22.2022. doi: 10.1523/ENEURO.0244-22.2022 (PMC9910575; doi:10.1523/ENEURO.0244-22.2022)
Supplement: Table 5-5 — Intragroup (Sham) comparison of Skeleton analysis in different regions of the cortex. One-way repeated-measures ANOVA followed by Tukey’s test. Colored cells indicate p-values < 0.05. Download Table 5-5, DOC file. [file enu-eN-MNT-0244-22-s11.doc]

| **Skeleton** | **Total Branches Length** | **Branches** | **Junctions** | **End-points** |
| --- | --- | --- | --- | --- |
| **IBZ_IL_-RZ_IL_** | 0.81091 | 0.33086 | 0.38224 | 0.19282 |
| **IBZ_IL_-IBZ_CL_** | 0.68952 | 0.28119 | 0.2935 | 0.27332 |
| **IBZ_IL_-IC_CL_** | 0.60919 | 0.17002 | 0.18659 | 0.15031 |
| **RZ_IL_-IBZ_CL_** | 0.99557 | 0.99923 | 0.9965 | 0.9938 |
| **RZ_IL_-IC_CL_** | 0.98177 | 0.95927 | 0.94653 | 0.99797 |
| **IBZ_CL_-IC_CL_** | 0.9989 | 0.98236 | 0.98655 | 0.97199 |
| **Skeleton** | **Junction Pixel** | **Average Branches Length** | **Maximum Branches Length** |  |
| **IBZ_IL_-RZ_IL_** | 0.43291 | 0.71809 | 0.99185 |  |
| **IBZ_IL_-IBZ_CL_** | 0.31615 | 0.5706 | 0.99952 |  |
| **IBZ_IL_-IC_CL_** | 0.24001 | 0.30705 | 0.99942 |  |
| **RZ_IL_-IBZ_CL_** | 0.99359 | 0.99329 | 0.99805 |  |
| **RZ_IL_-IC_CL_** | 0.96477 | 0.853 | 0.99828 |  |
| **IBZ_CL_-IC_CL_** | 0.99658 | 0.94745 | 1 |  |
