# Supplementary material for: Photothrombotic Middle Cerebral Artery Occlusion in Mice: A Novel Model of Ischemic Stroke
Source: eNeuro. 2023 Feb 7;10(2):ENEURO.0244-22.2022. doi: 10.1523/ENEURO.0244-22.2022 (PMC9910575; doi:10.1523/ENEURO.0244-22.2022)
Supplement: Table 5-6 — Intragroup (MCAPT) comparison of Skeleton analysis in different regions of the cortex. One-way repeated-measures ANOVA followed by Tukey’s test. Colored cells indicate p-values < 0.05. Download Table 5-6, DOC file. [file enu-eN-MNT-0244-22-s12.doc]

| **Skeleton** | **Total Branches Length** | **Branches** | **Junctions** | **End-points** |
| --- | --- | --- | --- | --- |
| **IBZ_IL_-RZ_IL_** | 0.00276 | 0.04385 | 0.0258 | 0.04458 |
| **IBZ_IL_-IBZ_CL_** | 0.1434 | 0.26307 | 0.0418 | 0.30829 |
| **IBZ_IL_-IC_CL_** | 0.03345 | 0.26655 | 0.98186 | 0.33687 |
| **RZ_IL_-IBZ_CL_** | 0.15225 | 0.69344 | 0.13353 | 0.63353 |
| **RZ_IL_-IC_CL_** | 0.5003 | 0.68818 | 0.84919 | 0.59533 |
| **IBZ_CL_-IC_CL_** | 0.82423 | 1 | 0.96399 | 0.99989 |
| **Skeleton** | **Junction Pixel** | **Avarage Branches Length** | **Maximum Branches Length** |  |
| **IBZ_IL_-RZ_IL_** | 0.06125 | 0.01006 | 0.14825 |  |
| **IBZ_IL_-IBZ_CL_** | 0.2695 | 0.99619 | 0.9455 |  |
| **IBZ_IL_-IC_CL_** | 0.36845 | 0.06826 | 0.07163 |  |
| **RZ_IL_-IBZ_CL_** | 0.7908 | 0.0069 | 0.33276 |  |
| **RZ_IL_-IC_CL_** | 0.66301 | 0.69652 | 0.97012 |  |
| **IBZ_CL_-IC_CL_** | 0.99549 | 0.04702 | 0.17521 |  |
